# Cobalt and Yttrium Doping to Activate Dual‐Site Mechanism of Amorphous NiFeOOH for Large‐Current Water Electrooxidation

**DOI:** 10.1002/advs.202512638

**Published:** 2025-09-08

**Authors:** Xiaojing Lv, Mingzhe Li, Hongye Qin, Lifang Jiao, Yuzhen Lv, Hongyun Luo, Wei Zhou, Lin Guo

**Affiliations:** ^1^ Hangzhou International Innovation Institute Beihang University Hangzhou 311115 China; ^2^ School of Chemistry Beihang University Beijing 100191 China; ^3^ School of Materials Science and Engineering Beihang University Beijing 100191 China; ^4^ School of Energy Power and Mechanical Engineering North China Electric Power University Beijing 102206 China; ^5^ Key Laboratory of Advanced Energy Materials Chemistry (Ministry of Education) Collaborative Innovation Center of Chemical Science and Engineering College of Chemistry Nankai University Tianjin 300071 China

**Keywords:** doping, dual‐site mechanism, kinetics, nickel‐iron, oxygen evolution reaction

## Abstract

The difference in hydroxyl adsorption between Ni and Fe sites in NiFeOOH limits the efficient dual‐site synergistic mechanism (DSSM) during oxygen evolution reaction (OER). Here, a novel needle‐array electrodeposition is reported for the scalable and efficient fabrication of Co and Y co‐doped NiFeOOH catalyst. It achieves an ultralow overpotential of 270 mV at 1 A cm^−2^ with a small Tafel slope of 30.7 mV dec^−1^. It maintains stable operation at 1 A cm^−2^ for 1500 hrs with 98 % initial potential retention. When integrated into a 25 cm^2^ anion exchange membrane electrolyzer, the system only needs 2.13 V to achieve 1 A cm^−2^. XPS, XAS, and DFT studies reveal that Co and Y dopants increase the Lewis acidity of Ni sites, enhancing ^*^OH adsorption. Concurrently, the incorporation of large Y atoms induces lattice distortion and elongates Fe─O─M bonds, weakening ^*^OH binding at Fe sites. This dual‐site modulation reduces adsorption disparity, activates NiFe dual‐active centers, and promotes the DSSM pathway, as confirmed by in situ ATR‐SEIRAS. The rate‐determining step energy is lowered to 1.71 eV, significantly outperforming the conventional AEM pathway (2.51 eV). This work provides dual‐site modulation into engineering high‐performance NiFe‐based OER catalysts for practical water electrolysis.

## Graphical abstract

The Co and Y‐doped amorphous NiFeOOH catalyst is synthesized via a novel needle‐array electrodeposition. The co‐doping tunes the adsorption energies at Ni and Fe sites, shifting the OER pathway from the traditional adsorbate evolution mechanism to a dual‐site synergistic mechanism confirmed by in situ ATR‐SEIRAS, thereby enhancing large‐current water electrooxidation kinetics.

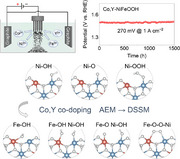

## 1 Introduction

NiFeOOH represents one of the most active catalytic systems for alkaline oxygen evolution reaction (OER) to date.^[^ ^1^ ^]^ Recent studies have revealed that NiFe sites not only serve as independent active centers, but also cooperate through a dual‐site synergistic mechanism in catalytic processes.^[^ ^2^ ^]^ In the four‐electron OER process, this dual‐site mechanism replaces the ^*^O→^*^OOH conversion step in the traditional adsorption evolution mechanism (AEM) into a ^*^O─O^*^ coupling between neighboring sites, thereby breaking the inherent linear scaling relationship of intermediate adsorption energies in single‐site catalysis.^[^ ^3^ ^]^ This unique reaction pathway reconstruction substantially lowers the energy barrier of the rate‐determining step (RDS), resulting in a significant enhancement in catalytic activity.^[^ ^4^ ^]^ Therefore, activating the dual‐site synergistic mechanism (DSSM) in NiFe‐based OER catalysts via targeted design strategies holds great promise for further performance improvement.

**Figure 1:**
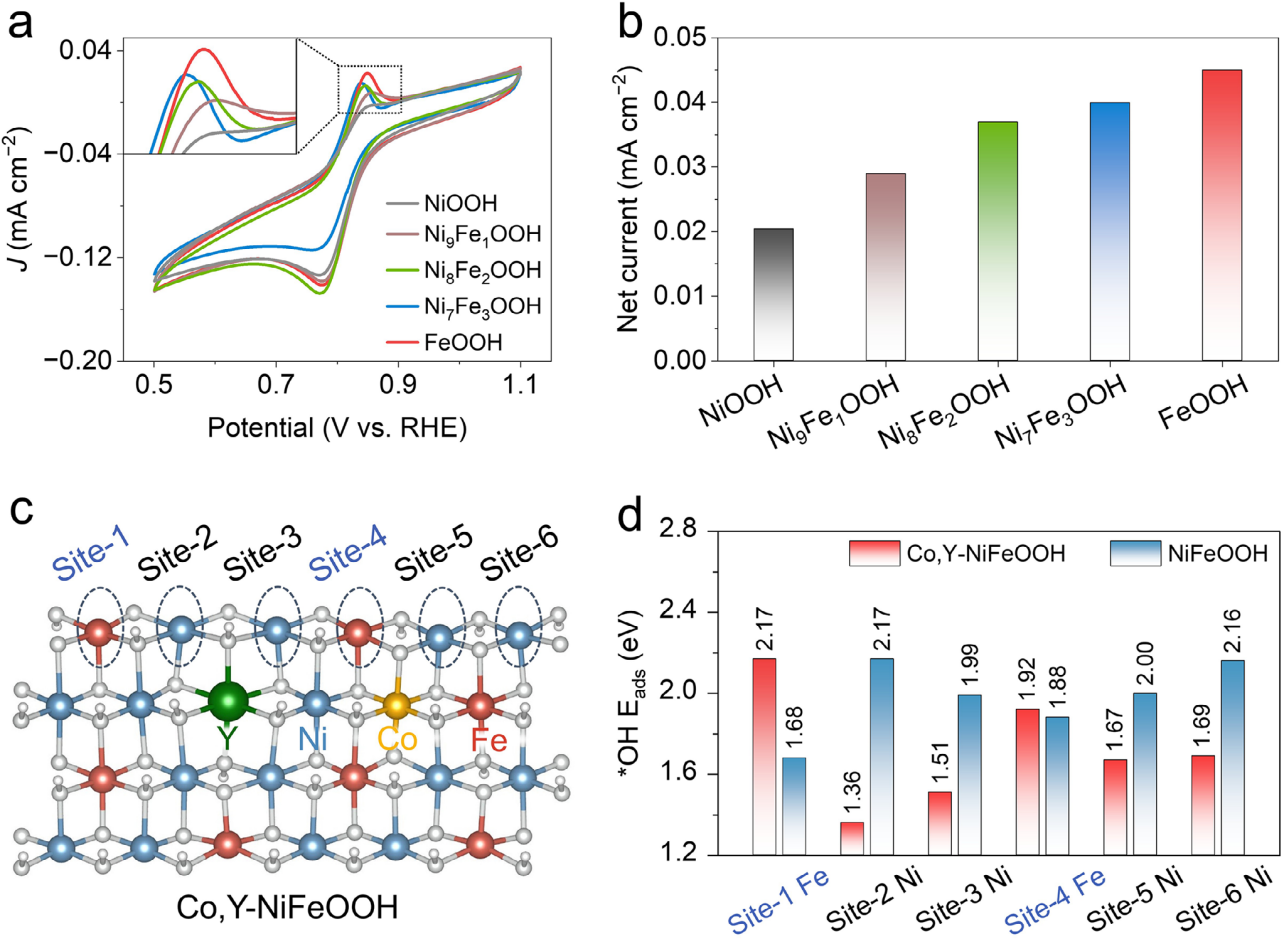
a) CV curves of NiOOH, Ni_9_Fe_1_OOH, Ni_8_Fe_2_OOH, Ni_7_Fe_3_OOH, and FeOOH in 1m KOH at a scan rate of 5 mV s^−1^. b) Corresponding comparison of the net current for oxidation peaks. c) Atomic model of Co,Y‐NiFeOOH with selected 6 adsorption sites toward hydroxyl. d) Comparison of adsorption energy toward *OH on Co,Y‐NiFeOOH, and NiFeOOH.

The DSSM imposes stricter geometric constraints on metal active sites due to its dependence on synchronous participation of adjacent metal sites.^[^ ^5^ ^]^ Only symmetric bimetallic sites with suitable atomic distances can facilitate ^*^O─O^*^ radical coupling with a low energy barrier.^[^ ^6^, ^7^ ^]^ For example, Wang et al. demonstrated that planar Fe─Co dual sites with optimal atomic spacing and stereochemical configuration synergistically promoted dehydrogenation to form O─O intermediates.^[^ ^8^ ^]^ Similarly, Zhang et al. constructed CoFeS_x_ nanoclusters on carbon nanotubes, where Co─Fe dual sites cooperatively generated Co─O─O─Fe intermediates, activating a segmented dual‐site mechanism.^[^ ^9^ ^]^ These studies confirm that adjacent six‐coordinated Co–O–Fe units satisfy the spatial requirements for DSSM. However, activating DSSM in an analogous Ni─O─Fe unit remains a fundamental challenge. That is, adjacent Ni and Fe sites must synchronously undergo ^*^OH adsorption and ^*^OH to ^*^O conversion, but the significant disparity in their ^*^OH adsorption energies leads to competitive adsorption and a higher overall energy barrier. The cyclic voltammetry (CV) results (**Figure** **1**a,b) also show that as the Fe content increases from NiOOH to FeOOH, the peak current density corresponding to ^*^OH adsorption progressively increases, confirming the significant difference in ^*^OH adsorption capability between Ni and Fe sites. Table S1 (Supporting Information) shows the molar ratios of elements in Ni_x_Fe_y_OOH.

Our group previously achieved balanced ^*^OH adsorption between Ni and Fe sites by Co doping in combination with introducing Cr vacancies into NiFeOOH.^[^ ^2^ ^]^ Lai et al. also reported co‐doping with Co and W, but their catalyst follows the AEM pathway and involves only the Ni sites.^[^ ^10^ ^]^ In an alkaline electrolyte, doped Cr and W readily convert to chromate and tungstate ions, respectively, forming metal vacancies.^[^ ^11^ ^]^ To further refine the aforementioned dual‐site engineering strategy, density functional theory (DFT) calculations were employed to identify substitutable elements as a rational alternative to uncontrollable metal vacancies. As shown in Figure 1c,d, a higher adsorption energy indicates less favorable ^*^OH adsorption. Additional atomic structures are presented in Figures S1 and S2 (Supporting Information). The results suggest that Co and Y co‐doping significantly enhances ^*^OH adsorption on Ni sites while simultaneously suppressing it on Fe sites. Therefore, we propose a needle‐array electrodeposition strategy to simultaneously achieve rapid mass production and one‐step Co and Y co‐doping. The resulting Co,Y‐NiFeOOH requires only 270 mV to achieve a large current density of 1 A cm^−2^ for OER in 1m KOH. It also maintains stable operation at 1 A cm^−2^ for 1500 hrs with 98 % retention of the initial potential. Even under high‐frequency unsteady‐state testing, it maintains over 80 % energy efficiency and stable performance for over 100 hrs without noticeable decay. Experimental and calculation results demonstrate that Co and Y co‐doping transforms NiFeOOH from the AEM catalytic pathway to the DSSM mechanism, leading to accelerated reaction kinetics.

## 2 Results and Discussion

**Figure 2:**
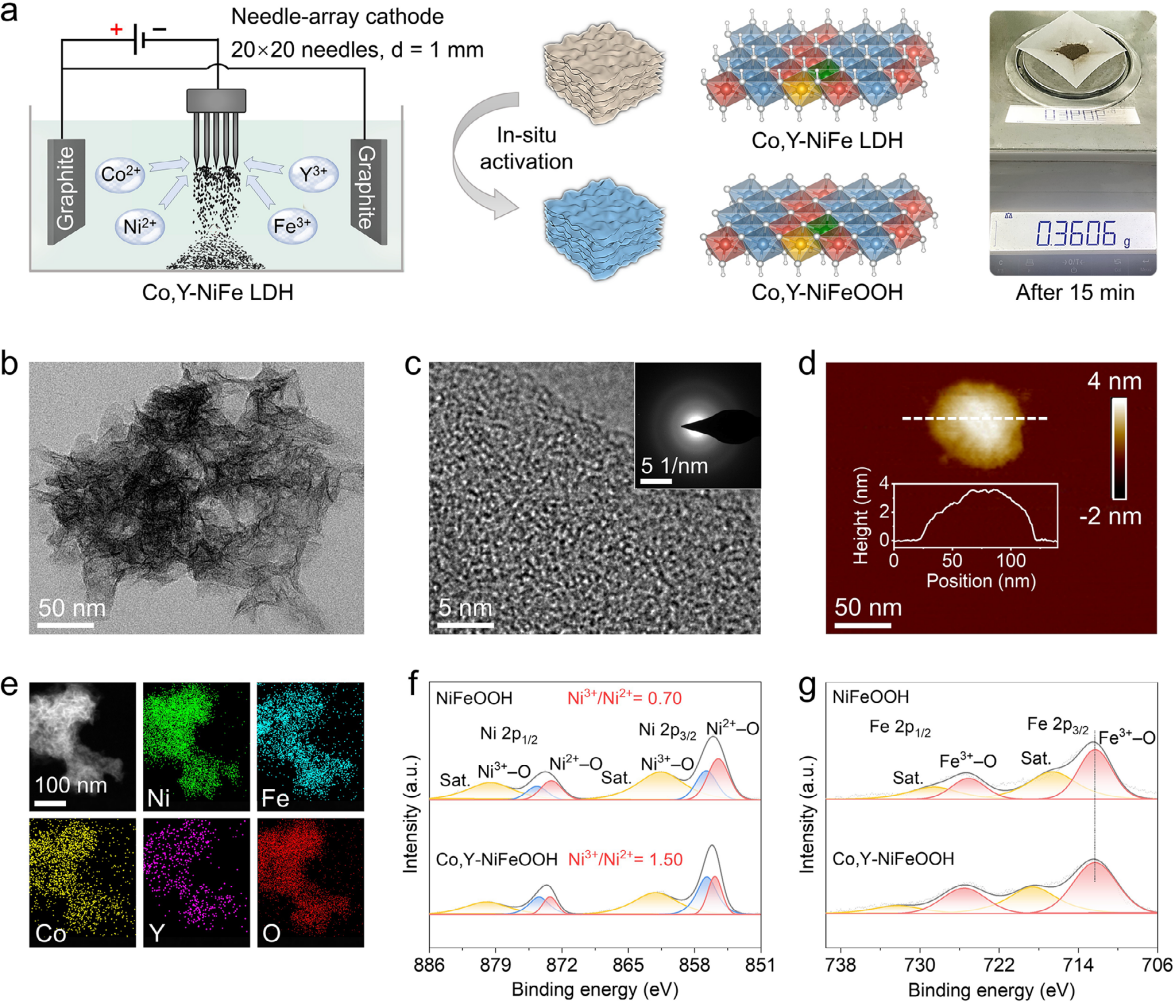
Synthesis and characterization of Co,Y‐NiFeOOH. a) Schematic illustration for the synthesis of Co,Y‐NiFeOOH by the needle‐array electrodeposition method. b) TEM image and c) HRTEM image of Co,Y‐NiFeOOH with SAED pattern inserted. d) AFM image and height profile of one nanosheet. e) EDS elemental mapping of Ni, Fe, Co, Y, and O from Co,Y‐NiFeOOH. f,g) High‐resolution XPS spectra of Ni 2p and Fe 2p of NiFeOOH and Co,Y‐NiFeOOH.

**Figure** **2** shows the synthetic process and characterizations of Co,Y‐NiFeOOH. As shown in Figure 2a, we used graphite as an anode and homemade needle arrays (1 mm in diameter, 400 needles) as a cathode. The metal salts were dissolved in the electrolyte. Metal cations migrate to the cathode and react on its surface to directly form Co and Y co‐doped NiFe LDH powder. Because of the special shape of the needle, the powder is prone to falling off the needle surface. Compared with hydrothermal and electrodeposition methods using substrates,^[^ ^12^, ^13^ ^]^ this one‐step electrodeposition method enables the continuous and large‐scale synthesis when increasing the reaction solution volume and the number of needles used. The precursor was then transformed to the Co,Y‐NiFeOOH catalyst by in‐situ electrochemical activation. For more synthetic details, see the Supporting Information. The TEM (Figure 2b) image reveals a nanoscale lamellar morphology. The HRTEM image and the corresponding SAED pattern confirm the amorphous nature of these sheets, which is further supported by the XRD pattern shown in Figure S3 (Supporting Information). The AFM image and height profile (Figure 2d) indicate that the amorphous sheets are ≈4 nm thick. EDS elemental mapping (Figure 2e) shows a uniform distribution of Ni, Fe, Co, and Y throughout the material. The elemental composition was further quantified by ICP‐OES (Table S2, Supporting Information), yielding a Ni:Fe:Co:Y molar ratio of ≈7:2:0.5:0.5 for Co,Y‐NiFeOOH, compared to a Ni:Fe ratio of 7:2 for undoped NiFeOOH. For comparison, characterization results of control samples are provided in Figure S4 (Supporting Information).

XPS analysis was conducted to investigate the influence of Co and Y co‐doping on the valence states of Ni and Fe in NiFeOOH. As shown in the Ni 2p XPS spectra (Figure 2f), the peaks at 855.9 and 873.3 eV correspond to Ni 2p_3/2_ and Ni 2p_1/2_ of Ni^2+^─O, respectively, while the peaks at 856.9 and 874.3 eV are attributed to Ni 2p_3/2_ and Ni 2p_1/2_ of Ni^3+^─O.^[^ ^14^ ^]^ The Ni^3+^/Ni^2+^ ratio increases from 0.70 in pristine NiFeOOH to 1.50 in Co,Y‐NiFeOOH, indicating a substantial increase in the Ni oxidation state as a result of Co and Y co‐doping. For the Fe 2p XPS spectra (Figure 2g), the peaks at 712.3 and 725.6 eV are assigned to Fe 2p_3/2_ and Fe 2p_1/2_ of Fe^3+^─O, respectively.^[^ ^15^ ^]^ The absence of a noticeable peak shift suggests that the valence state of Fe remains largely unchanged upon doping.

**Figure 3:**
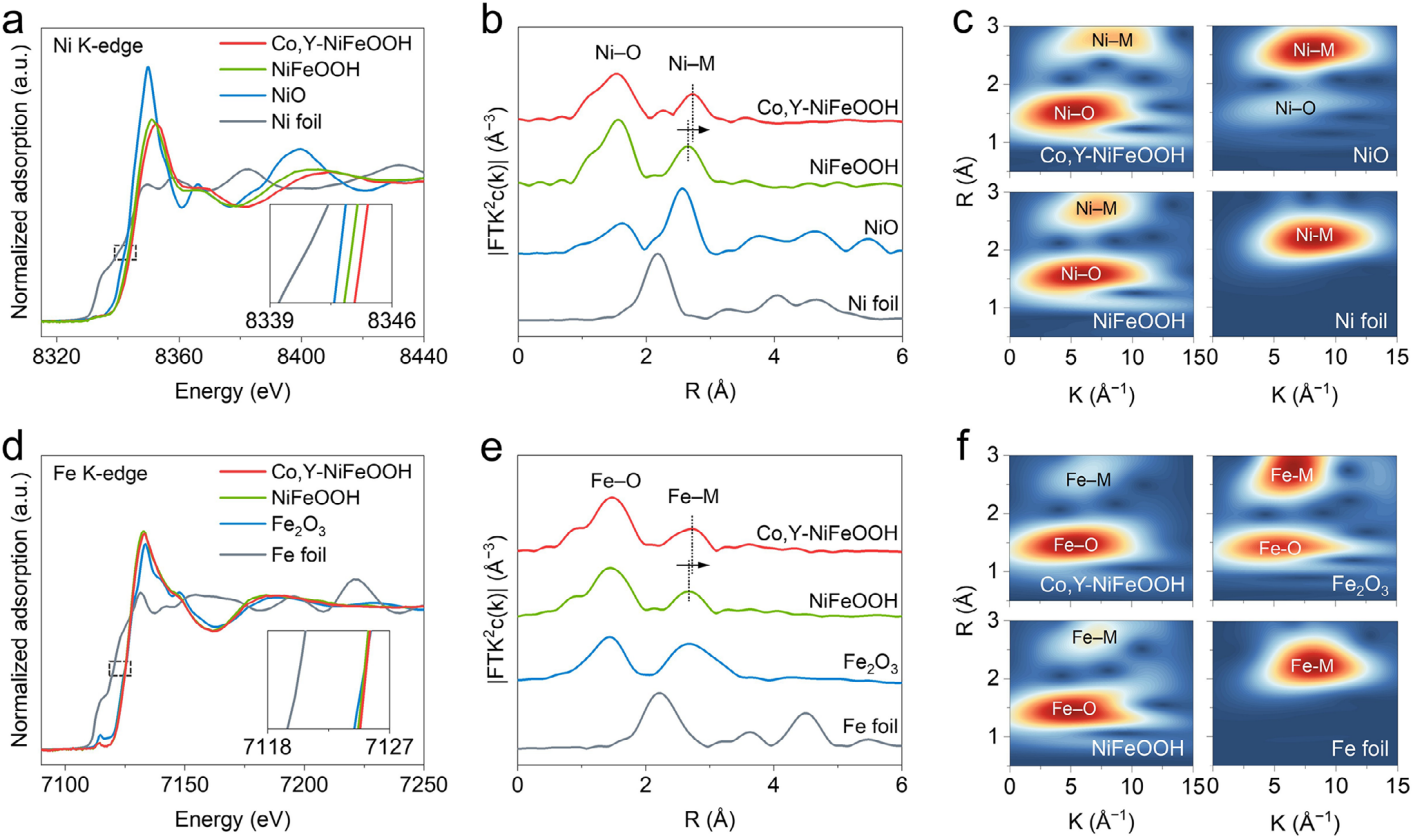
Electronic structure optimization on Ni‐Fe dual sites by Co and Y doping. a) Ni K‐edge XANES spectra with a magnified marked region inserted. b) Fourier‐transform spectra from EXAFS at Ni K‐edge. c) Corresponding WT‐EXAFS plots. NiO and Ni foil were used for reference. d) Fe K‐edge XANES spectra with a magnified marked region inserted. e) Fourier‐transform spectra from EXAFS at Fe K‐edge. f) Corresponding WT‐EXAFS plots. Fe_2_O_3_ and Fe foil were used for reference.

Further insights into the refined valence state changes were obtained through X‐ray absorption spectroscopy (XAS). Specifically, X‐ray absorption near‐edge structure (XANES) and wavelet transform extended X‐ray absorption fine structure (WT‐EXAFS) analyses were conducted to disclose the atomic‐level effects of Co and Y co‐doping on the electronic and coordination environment of NiFeOOH. The Ni K‐edge XANES spectra (**Figure** **3**a) show that the absorption edges of both Co,Y‐NiFeOOH, and NiFeOOH are located to the right of NiO, indicating that the oxidation state of Ni exceeds +2. Upon Co and Y co‐doping, the Ni K‐edge shifts further to higher energies, revealing an increase in the Ni oxidation state. Notably, the Ni─O bond position remains unchanged, suggesting that the local oxygen coordination environment around Ni is preserved (Figure 3b). Meanwhile, the Ni─M bond shifts to a longer bond length, as evidenced by its rightward movement in R‐space, indicating elongation of the Ni─Co/Ni─Y bonds.^[^ ^16^ ^]^ This elongation might be attributed to the larger ionic radius of the dopants and altered electronic interactions within the doped lattice.^[^ ^17^, ^18^ ^]^ The WT‐EXAFS analysis of Co,Y‐NiFeOOH (Figure 3c) shows a slight upshift for Ni─M in the R‐space region compared to NiFeOOH, reinforcing the observation of bond elongation. The Fe K‐edge XANES spectra (Figure 3d) indicate that Co and Y co‐doping does not significantly affect the valence state of Fe, which remains near +3. However, the bond length of Fe─M in the second coordination shell increases (Figure 3e,f). Structural parameters extracted from EXAFS fitting also reveal an increase in both Ni─M and Fe─M bond length (Table S3, Supporting Information). Additionally, the Co K‐edge XANES spectra (Figure S5, Supporting Information) reveal that the absorption edge of Co,Y‐NiFeOOH, lies to the right of CoO, suggesting that the oxidation state of Co also exceeds +2. The XPS results (Figure S6a,b, Supporting Information) also support the obvious increase in the oxidation state of Ni and the unobvious change in the oxidation state of Fe. Specifically, the doped Co raises the oxidation state of Ni while slightly reducing that of Fe, whereas the doped Y increases the oxidation states of Ni, Fe, and even the doped Co. Through their synergistic effect, Ni undergoes a significant increase in valence state, while Fe retains an almost unchanged oxidation state. It can be concluded that doped Co, especially Y atoms, exhibit strong abilities to accept electrons, which can increase the Lewis acidity of Ni and thus strengthen the adsorption ability of Ni sites toward hydroxyl. The differential charge density plot in Figure S6e (Supporting Information) also confirms the electron consumption state around Co and Y, showing their strong electron‐withdrawing ability. Although the oxidation state of Fe remains largely unchanged, EXAFS data reveal that the Fe─O─M bonds are elongated due to the large ionic radius of the doped Y atoms, which can weaken the hydroxyl adsorption capability of the Fe sites. Given that ^*^OH adsorption is sensitive to the oxidation states of Ni and Fe, these findings align with DFT results (Figure 1c,d), demonstrating that Co and Y co‐doping effectively narrows the adsorption strength of ^*^OH at Ni and Fe sites, optimizing the catalytic behavior.

**Figure 4:**
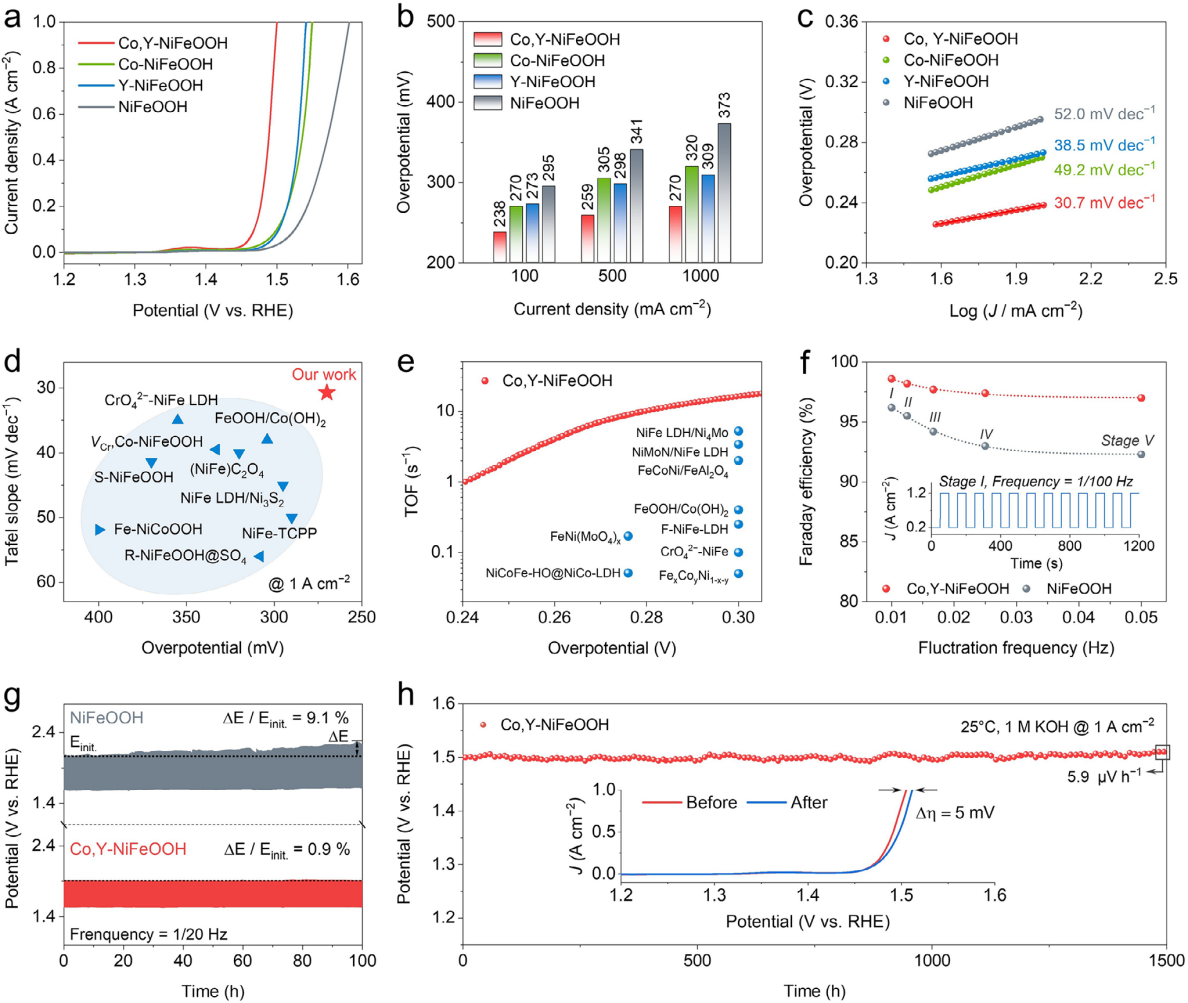
Electrochemical OER performance. a) LSV curves with iR correction. b) Comparison of overpotential values at 100, 500, and 1000 mA cm^−2^. c) Tafel slopes of Co,Y‐NiFeOOH, Co‐NiFeOOH, Y‐NiFeOOH, and NiFeOOH. d) Comparison of Tafel slopes and overpotentials with recently reported catalysts. e) TOF‐value plot of Co,Y‐NiFeOOH in comparison to recently reported NiFe‐based catalysts. f) Faraday efficiency at five stages using Co,Y‐NiFeOOH, and NiFeOOH. Five unstable types of rectangular waves were employed as signal input to simulate a fluctuating state with a range from 20 to 120 % based on the fixed current density of 1 A cm^−2^. g) Stability test of Co,Y‐NiFeOOH, and NiFeOOH under fluctuating state of stage V. h) Stability test of Co,Y‐NiFeOOH using chronopotentiometry at 1 A cm^−2^. The inset shows LSV curves before and after testing.

The OER performance of the as‐prepared catalysts was evaluated using a standard three‐electrode setup in 1m KOH. The linear sweep voltammetry (LSV) curves in **Figure** **4**a show that Co,Y‐NiFeOOH exhibits overpotentials of 238 and 270 mV at current densities of 100 and 1000 mA cm^−2^, respectively. LSV curves without iR correction are shown in Figure S7 (Supporting Information). As shown in Figure 4b, these values are significantly lower than those of Co‐NiFeOOH (270 and 320 mV), Y‐NiFeOOH (273 and 309 mV), and NiFeOOH (295 and 373 mV) accordingly. Besides, Co,Y‐NiFeOOH shows a Tafel slope of 30.7 mV dec^−1^, which is markedly lower than the values for Co‐NiFeOOH (49.2 mV dec^−1^), Y‐NiFeOOH (38.5 mV dec^−1^), and NiFeOOH (52.0 mV dec^−1^). This suggests a significant enhancement in reaction kinetics resulting from the co‐doping of Co and Y. As summarized in Figure 4d and Table S4 (Supporting Information), Co,Y‐NiFeOOH demonstrates competitive overpotential values at 1000 mA cm^−2^ and a favorable Tafel slope in comparison with other recently reported NiFe‐based hydroxide or oxyhydroxide catalysts.^[^ ^2^, ^16^, ^19^, ^20^, ^21^, ^22^, ^23^, ^24^, ^25^ ^]^ The electrochemical surface area (ECSA) was used to calculate the turnover frequency, which quantifies the number of oxygen molecules generated per active site per second, as shown in Figure 4e. A turnover frequency of 16.2 s^−1^ was recorded at 1.53 V versus RHE, outperforming many excellent NiFe‐based catalysts. ^[^ ^13^, ^23^, ^24^, ^25^, ^26^, ^27^, ^28^, ^29^, ^30^ ^]^ Co,Y‐NiFeOOH also exhibits outstanding mass activity (Figure S8, Supporting Information) and demonstrates superior TOF values (Figure S9, Supporting Information).

Co,Y‐NiFeOOH also demonstrates the highest ECSA‐normalized current density of 8.1 mA cm^−2^_ECSA_ at an overpotential of 270 mV (Figure S10, Supporting Information), indicating excellent intrinsic activity. Electrochemical impedance spectroscopy (EIS) measurements at 1.47 V confirm the improved reaction kinetics, with Co,Y‐NiFeOOH showing a very low charge transfer resistance (R_ct_) of 0.86 Ω. This is significantly lower than the values observed for Co‐NiFeOOH at 3.60 Ω, Y‐NiFeOOH at 2.86 Ω, and NiFeOOH at 6.98 Ω, as shown in Figure S11 (Supporting Information). To further assess the practical applicability of the catalyst, the Faradaic efficiency was measured at a current density of 1 A cm^−2^ (Figure S12, Supporting Information). Co,Y‐NiFeOOH exhibits the highest Faraday efficiency of 98.5 %, whereas NiFeOOH demonstrates a value of 95.7 %.

Given the intermittent nature of renewable energy sources such as wind and solar power, evaluating OER performance under unsteady‐state conditions is essential for assessing the practical viability of catalytic materials. As shown in Figure 4f and Figure S13 (Supporting Information), rectangular current waveforms ranging from 0.2 to 1.2 A cm^−2^ were applied to simulate power fluctuations from 20 to 120 %. A five‐stage protocol was developed to systematically explore the frequency‐dependent performance. Specifically, step durations of 100, 80, 60, 40, and 20 s were implemented, corresponding to equivalent frequencies of 1/100, 1/80, 1/60, 1/40, and 1/20 Hz, respectively. The fitted curves clearly indicate that higher frequency disturbances lead to reduced Faraday efficiency. Notably, Co,Y‐NiFeOOH consistently exhibits superior Faraday efficiency above 92 % compared to NiFeOOH at all stages. The original measured data for Co,Y‐NiFeOOH, and NiFeOOH are provided in Figure S14 and Table S5 (Supporting Information). An accelerated aging test was conducted under the most extreme conditions at a frequency of 1/20 Hz to evaluate the long‐term durability of Co,Y‐NiFeOOH, and NiFeOOH (Figure 4g). After 100 hrs of testing, Co,Y‐NiFeOOH displays a minimal performance decay of 0.9 % compared to a much higher decay of 9.1 % for NiFeOOH. To further examine stability, Co,Y‐NiFeOOH was operated continuously for 1500 h at a high current density of 1 A cm^−2^. During this prolonged operation, the potential increases at a remarkably low rate of 5.9 µV h^−1^ (Figure 4h). A comparison of the LSV curves before and after stability testing shows that the overpotential increased by only 5 mV at 1 A cm^−2^, corresponding to a performance retention of 98.2 %. The TEM and ICP results collectively demonstrate the stability of the catalyst (Figure S15 and Table S6, Supporting Information). This comprehensive assessment of OER activity and stability under both steady‐state and unsteady‐state conditions confirms the excellent practical performance of Co,Y‐NiFeOOH.

**Figure 5:**
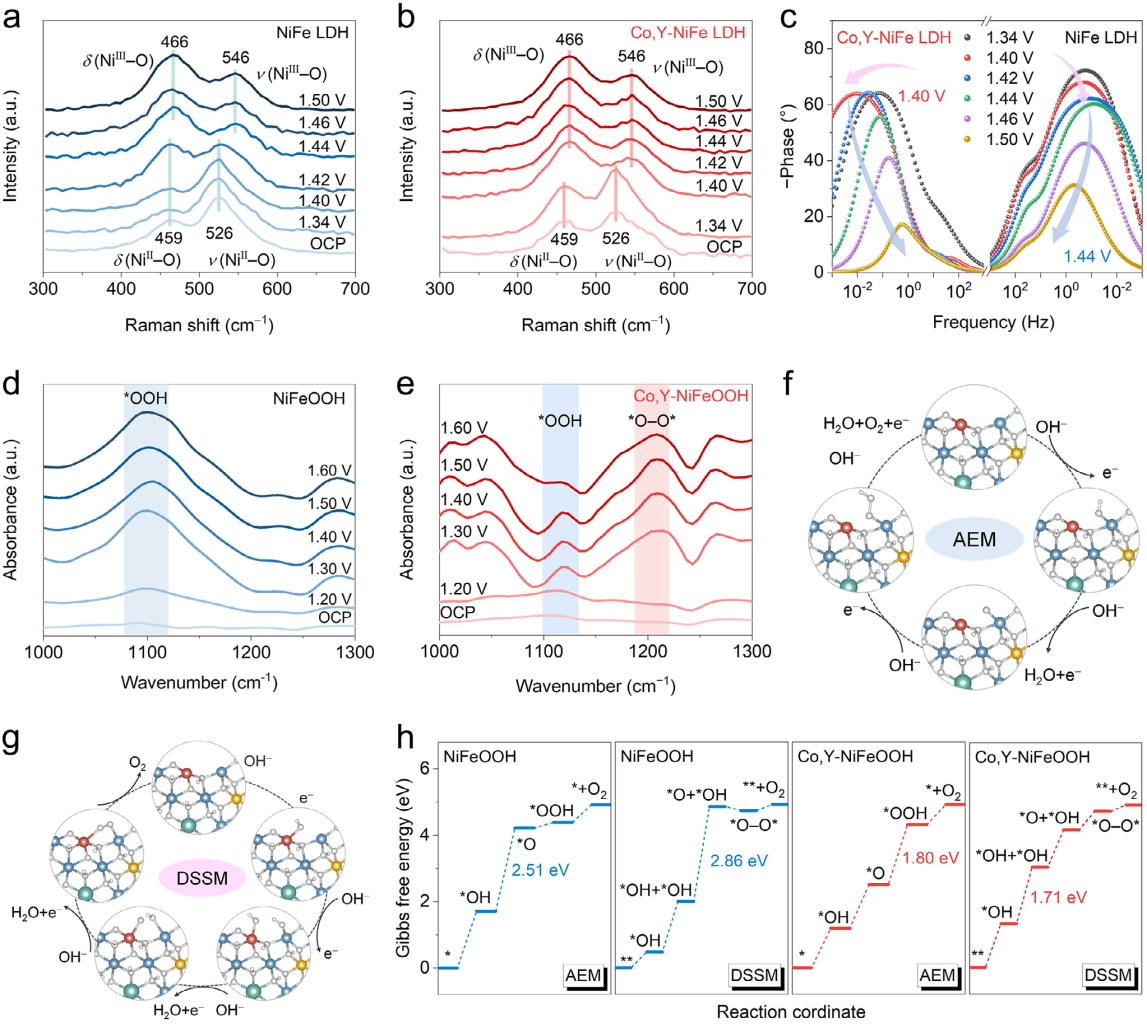
Mechanism investigation by experiments and DFT calculations. In situ Raman spectra of a) NiFe LDH and b) Co,Y‐NiFe LDH in the OER process. c) Bode plots for Co,Y‐NiFe LDH, and NiFe LDH tested by in‐situ EIS. In situ ATR‐SEIRAS spectra of d) NiFeOOH and e) Co,Y‐NiFeOOH, disclosing the activated dual‐site mechanism of Co,Y‐NiFeOOH. Schematic illustrations of f) AEM and g) DSSM pathways. h) Gibbs free energy diagrams during AEM and DSSM pathways of NiFeOOH and Co,Y‐NiFeOOH.

To further elucidate the mechanism by which Co and Y co‐doping enhances OER performance, in situ experiments and DFT calculations were conducted. As mentioned earlier, the NiFe LDH precursor powder was first synthesized and subsequently transformed into NiFeOOH catalysts through in situ electrochemical activation. Hence, a rapid activation process results in energy savings. In situ Raman spectroscopy reveals that the *δ*(Ni─O) and *ν*(Ni─O) bands of NiFe LDH shift from 459 to 466 cm^−1^ and from 526 to 546 cm^−1^ at 1.44 V (**Figure** **5**a). In comparison, Co,Y‐NiFe LDH exhibits similar band shifts at a lower potential of 1.40 V, as illustrated in Figure 5b. This result indicates that Co and Y co‐doping accelerates the self‐activation process of NiFe LDH. Subsequently, in situ EIS measurements were performed to validate this acceleration. As shown in Figure 5c and Figure S16 (Supporting Information), the high‐frequency region of the Bode plots corresponds to electro‐oxidation processes of the catalytic materials, while the low‐frequency region reflects the OER process.^[^ ^31^, ^32^ ^]^ Co,Y‐NiFe LDH exhibits the lowest initial OER potential of 1.40 V, outperforming Co‐NiFe LDH at 1.42 V, Y‐NiFe LDH at 1.42 V, and pristine NiFe LDH at 1.44 V. These observations are consistent with the in situ Raman results. The XPS spectra of the as‐prepared samples before and after activation, further elucidate a higher degree of activation by Co and Y co‐doping (Figure S17, Supporting Information). The Nyquist plots from in situ EIS (Figure S18, Supporting Information) further show that Co,Y‐NiFe LDH displays significantly lower R_ct_ than the control samples across the full range of applied potentials, both in the electro‐oxidation and OER regions. This finding confirms that Co and Y co‐doping synergistically facilitates electron transfer, thus improving OER kinetics.

To investigate the OER reaction pathway, in situ attenuated total reflection surface‐enhanced infrared absorption spectroscopy (ATR‐SEIRAS) was employed to track potential‐dependent evolution of reaction intermediates.^[^ ^6^, ^33^ ^]^ NiFeOOH exhibits a clear ^*^OOH signal at 1100 cm^−1^, as shown in Figure 5d, indicating its primary reliance on the AEM pathway.^[^ ^34^ ^]^ In contrast, Co,Y‐NiFeOOH presents two prominent peaks at 1208 and 1117 cm^−1^, which correspond to the O─O stretching vibration of ^*^O─O^*^ intermediates associated with the DSSM pathway and the ^*^OOH bending mode of the AEM pathway, respectively (Figure 5e).^[^ ^35^, ^36^ ^]^ Notably, the intensity of the O─O peak increases with applied potential, while the ^*^OOH signal diminishes, indicating a potential‐driven shift toward DSSM dominance in Co,Y‐NiFeOOH. These findings indicate that NiFeOOH predominantly follows an AEM pathway, whereas Co,Y‐NiFeOOH undergoes a DSSM‐dominated oxygen evolution.

DFT calculations were conducted to compare the theoretical Gibbs free energy barriers along both AEM and DSSM pathways for Co,Y‐NiFeOOH, Co‐NiFeOOH, Y‐NiFeOOH, and NiFeOOH. Their optimized atomic structures are presented in Figure S19 (Supporting Information). Figure 5f,g illustrates the evolution of reaction intermediates along AEM and DSSM pathways using Co,Y‐NiFeOOH as a representative example.^[^ ^37^ ^]^ For NiFeOOH, the calculated ΔG_RDS_ is 2.51 eV along the AEM pathway and 2.86 eV along the DSSM pathway (Figure S20, Supporting Information). Similar trends are observed in Co‐NiFeOOH with AEM at 1.98 eV and DSSM at 2.37 eV, and in Y‐NiFeOOH with AEM at 2.29 eV and DSSM at 2.70 eV (Figure S21, Supporting Information). Remarkably, Co,Y‐NiFeOOH reverses this trend, displaying a lower ΔG_RDS_ of 1.71 eV via the DSSM pathway compared to 1.80 eV via the AEM pathway (Figure 5h). This result represents the lowest energy barrier among all samples and highlights the enhanced OER kinetics of Co,Y‐NiFeOOH through the DSSM mechanism. Overall, these investigations demonstrate that the co‐doping of Co and Y not only promotes the self‐activation of NiFeOOH but also drives a mechanistic transition from AEM to DSSM, thereby enhancing OER kinetics.

**Figure 6:**
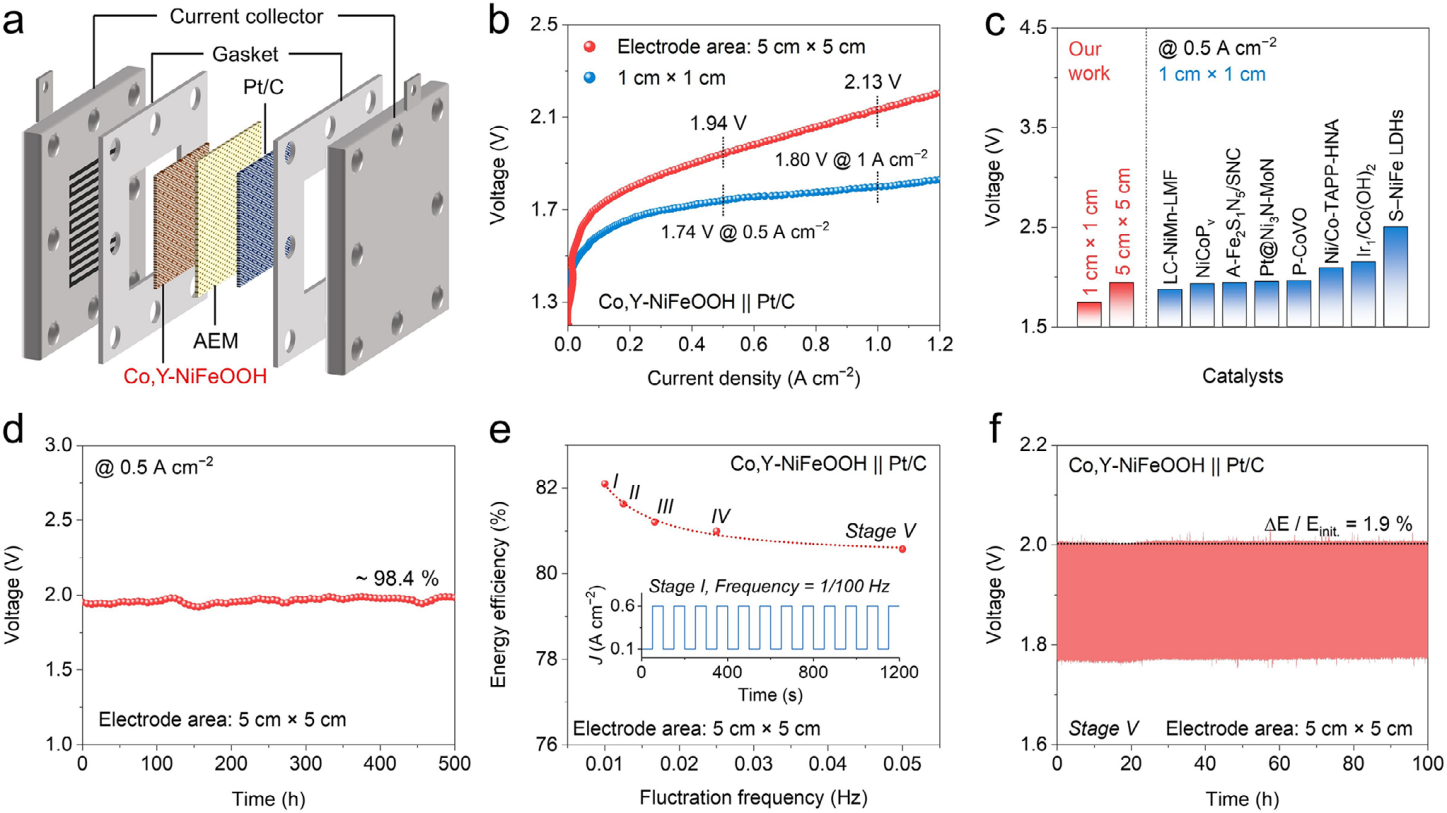
Performance of the AEMWE device based on Co,Y‐NiFeOOH. a) Schematic assembly of an AEMWE device. b) LSV curves measured in AEM devices using our catalyst with different electrode areas. c) Comparison of the voltage values at 0.5 A cm^−2^ with those recently reported using 1 cm × 1 cm electrodes. d) Durability test of the as‐obtained device at 0.5 A cm^−2^ for 500 hrs. e) Energy conversion efficiency at five stages of Co,Y‐NiFeOOH. Five types of rectangular waves were employed as signal input to simulate fluctuating state with the range from 20 to 120 % based on a fixed current density of 0.5 A cm^−2^. f) Stability test of Co,Y‐NiFeOOH under the fluctuating state of stage V.

To further assess its practical applicability, we assembled an anion exchange membrane (AEM) electrolyzer using a Co,Y‐NiFeOOH anode and a Pt/C cathode (**Figure** **6**a, details see the Supporting Information). The device requires only 1.74 and 1.94 V to reach a high current density of 0.5 A cm^−2^, and just 1.80 and 2.13 V to reach 1 A cm^−2^ for electrode areas of 1 and 25 cm^2^, respectively (Figure 6b). A comparison of the performance of 1 cm × 1 cm and 5 cm × 5 cm electrodes with recently reported devices (1 cm × 1 cm electrodes) is shown in Figure 6c and Table S7 (Supporting Information).^[^ ^31^, ^38^, ^39^, ^40^, ^41^, ^42^, ^43^, ^44^ ^]^ Notably, the electrolyzer achieves low operating voltages at 0.5 A cm^−2^, highlighting its efficiency. Moreover, the 25 cm^2^ electrode exhibits excellent durability, maintaining 98.4 % of its initial performance over 500 h at 0.5 A cm^−2^ (Figure 6d).

The applicability of the 25 cm^2^ device was further evaluated under fluctuating load conditions. As shown in Figures S22 and S23 (Supporting Information), rectangular current waveforms ranging from 0.1 to 0.6 A cm^−2^ were applied to simulate 20–120 % power fluctuations. Fluctuation frequencies of 1/100, 1/80, 1/60, 1/40, and 1/20 Hz were adopted, corresponding to five stages to represent increasingly severe fluctuation states. The fitted curves clearly show that higher‐frequency disturbances result in decreased energy conversion efficiency. For the Co,Y‐NiFeOOH || Pt/C system, the energy conversion efficiency remains above 80 % across all stages, meeting the International Energy Agency (IEA) target for the 2020–2050 period (Figure 6e).^[^ ^45^ ^]^ An accelerated aging test was also conducted under the harshest conditions with a fluctuation frequency of 1/20 Hz to assess the long‐term stability of the Co,Y‐NiFeOOH || Pt/C system (Figure 6f). After 100 h of continuous testing, it exhibited a minimal performance decay rate of only 1.9 %. Steady‐state and unsteady‐state tests that mimic real‐world wind and solar power conditions show that Co,Y‐NiFeOOH delivers both excellent kinetics and strong stability, highlighting its potential for practical application.

## 3 Conclusion

In summary, we report a novel needle‐array electrodeposition strategy for the scalable and efficient fabrication of Co and Y co‐doped NiFeOOH catalyst. The optimized material exhibits outstanding OER performance, requiring a low overpotential of just 270 mV to achieve a current density of 1 A cm^−2^ along with a small Tafel slope of 30.7 mV dec^−1^. It also demonstrates remarkable long‐term durability, maintaining 98 % of its initial performance after 1500 h of continuous operation at 1 A cm^−2^. Furthermore, a 25 cm^2^ AEM electrolyzer assembled with Co,Y‐NiFeOOH as the anode delivers 1 A cm^−2^ at a low cell voltage of 2.13 V. Even under high‐frequency dynamic operation, the system maintains an energy conversion efficiency above 80 % and exhibits negligible degradation over 100 h of operation. XPS, XAS, and DFT calculations reveal that Co and, particularly, Y dopants possess strong electron‐accepting capabilities. This increases the Lewis acidity of neighboring Ni centers and thus enhances hydroxyl adsorption at Ni sites. EXAFS analysis further shows that the incorporation of large Y atoms elongates Fe─O─M bonds, thereby weakening hydroxyl adsorption at Fe sites. This dual‐site electronic modulation enabled by Co and Y co‐doping reduces the disparity in *OH adsorption between Ni and Fe sites, activates the NiFe dual‐active centers, and promotes the DSSM pathway for the OER process, as confirmed by in situ ATR‐SEIRAS measurements, ultimately enhancing the catalytic kinetics. Accordingly, the calculated Gibbs free energy of the RDS is significantly reduced to 1.71 eV for Co,Y‐NiFeOOH, compared to 2.51 eV via the conventional AEM pathway in undoped NiFeOOH. This work offers critical insights and design principles for harnessing DSSM pathways in NiFe‐based OER catalysts, paving the way for their practical deployment in water electrolysis technologies.

## Conflict of Interest

The authors declare no conflict of interest.

## Supporting information



Supporting Information

## Data Availability

The data that support the findings of this study are available from the corresponding author upon reasonable request.
