# Supplementary material for: Cobalt and Yttrium Doping to Activate Dual‐Site Mechanism of Amorphous NiFeOOH for Large‐Current Water Electrooxidation
Source: Adv Sci (Weinh). 2025 Sep 8;12(45):e12638. doi: 10.1002/advs.202512638 (PMC12677668; doi:10.1002/advs.202512638)
Supplement: Supplementary file 1 — Supporting Information [file ADVS-12-e12638-s001.docx]

**Supporting Information**

**Cobalt and Yttrium Doping to Activate Dual-Site Mechanism of Amorphous NiFeOOH for Large-Current Water Electrooxidation**

*Xiaojing Lv^#^, Mingzhe Li^#^, Hongye Qin, Lifang Jiao*, Yuzhen Lv*, Hongyun Luo, Wei Zhou*, and Lin Guo*

X. Lv, Prof. W. Zhou, Prof. L. Guo

Hangzhou International Innovation Institute, Beihang University, Hangzhou 311115, China; School of Chemistry, Beihang University, Beijing 100191, China

E-mail: zhouwei@buaa.edu.cn

X. Lv, Prof. H. Luo

School of Materials Science and Engineering, Beihang University, Beijing 100191, China

M. Li, Prof. Y. Lv

School of Energy, Power and Mechanical Engineering, North China Electric Power University, Beijing 102206, China

E-mail: yzlv@ncepu.edu.cn

H. Qin, Prof. L. Jiao

Key Laboratory of Advanced Energy Materials Chemistry (Ministry of Education), Collaborative Innovation Center of Chemical Science and Engineering, College of Chemistry, Nankai University, Tianjin 300071, China.

E-mail: jiaolf@nankai.edu.cn

^#^The authors contributed equally.

**1.** **Experimental section**

***Materials and chemicals*.** Nickel(II) chloride hexahydrate (NiCl_2_·6H_2_O), iron(III) nitrate nonahydrate (Fe(NO_3_)_3_·9H_2_O), cobalt(II) chloride hexahydrate (CoCl_2_·6H_2_O), yttrium(III) nitrate hexahydrate (Y(NO_3_)_3_·6H_2_O), potassium hydroxide (KOH, 99.5 %) and ethanol (> 99.5 %) were purchased from Aladdin Co., Ltd. (Shanghai, China). Nickel foams (NF) were purchased from the Longshengbao Electronic Materials Business Department located in Yushan Town, Kunshan City, China. Nafion solution (5 wt%) was purchased from alfa Co., Ltd. The anion exchange membrane (Sustainion X37-50 grade RT) was provided by Dioxide Materials (Florida, United States). All aqueous solutions used in the experiments were prepared using ultrapure water (resistivity of 18.2 MΩ·cm, Milli-Q system). All chemicals were used without further purification.

*Synthesis of Co,Y-NiFeOOH*. First, the precursor powder of Co,Y-NiFe LDH layered double hydroxide (LDH) was synthesized via a one-step electrodeposition method by our homemade device. In this setup, two high-purity graphite plates served as the anode, while a square array electrode consisting of 400 needles (20 × 20) functioned as the cathode. Each needle had a diameter of 1 mm, with a center-to-center spacing of 1.5 mm. The electrolyte solution was prepared by dissolving 90 mM NiCl_2_·6H_2_O, 25 mM Fe(NO_3_)_3_·9H_2_O, 15 mM CoCl_2_·6H_2_O and 5 mM Y(NO_3_)_3_·6H_2_O in deionized (DI) water. During the electrodeposition, the needle tips were immersed 2 mm below the electrolyte surface, and a constant current of 0.2 A was applied. The resulting Co,Y-NiFe LDH powder continuously formed at the needle tips and precipitated to the bottom of the electrolytic cell. The collected powder was washed several times with ethanol and DI water to remove impurities. Finally, Co,Y-NiFeOOH was obtained through in situ electrochemical activation using chronopotentiometry at 100 mA cm^−2^ for 20 min.

***Synthesis of Co-NiFeOOH*****.** The process followed the same synthetic procedure as that of Co,Y-NiFeOOH, except that yttrium nitrate was omitted during the electrodeposition step.

***Synthesis of Y-NiFeOOH*.** The process generally followed the same synthetic procedure as that of Co,Y-NiFeOOH, except that cobalt chloride was excluded during the electrodeposition step.

***Synthesis of NiFeOOH*.** The process generally followed the synthetic procedure of Co,Y-NiFeOOH, except that both cobalt chloride and yttrium nitrate were omitted during the electrodeposition step.

***Synthesis of Ni_x_Fe_y_OOH, NiOOH, and FeOOH.*** The process generally followed the synthetic procedure of NiFeOOH, by employing different Ni/Fe molar ratios during the electrodeposition step. The electrolytes for Ni_9_Fe_1_OOH, Ni_8_Fe_2_OOH, and Ni_7_Fe_3_OOH were respectively prepared by dissolving 103.5/11.5 mM, 92/23 mM, and 80.5/34.5 mM of NiCl_2_·6H_2_O/Fe(NO_3_)_3_·9H_2_O in DI water. For NiOOH and FeOOH, the electrolyte was prepared by separately dissolving 115 mM NiCl_2_·6H_2_O or 115 mM Fe(NO_3_)_3_·9H_2_O in DI water.

***Material characterizations*:** The morphology and structure of as-synthesized samples were characterized by transmission electron microscopy (TEM, FEI Tecnai G2 F30), and atomic force microscopy (AFM, Bruker Dimension Icon). X-ray absorption fine structure (XAFS) spectra at the K-edges were collected at the BL16U1 beamline of the Shanghai Synchrotron Radiation Facility (SSRF, 3.5 GeV, 250 mA). All XAFS measurements were conducted at room temperature. Samples were pelletized into 13 mm diameter, 1 mm thick disks using graphite powder as a binder. X-ray diffraction (XRD) patterns were recorded on a Shimadzu XRD-6000 diffractometer using Cu-*K*_α_ radiation (λ=1.5416 Å). X-ray photoelectron spectroscopy (XPS) was performed using a ThermoFisher ESCALAB 250Xi spectrometer equipped with a monochromatic Al *K*_α_ X-ray source (150 W). All XPS spectra were calibrated to the C 1s peak. Elemental compositions (Ni, Fe, Co, and Y) were quantified using inductively coupled plasma optical emission spectroscopy (ICP-OES, Agilent 5110).

***Electrochemical experiments*:** Electrochemical measurements were conducted using a Gamry electrochemical workstation (Gamry Reference 1000, Gamry Instruments, USA). A conventional three-electrode setup was employed, comprising a nickel foam (1 cm × 1 cm) as the working electrode, a Hg/HgO electrode (in 1M KOH) as the reference electrode, and a platinum sheet as the counter electrode. To prepare the catalyst ink, approximately 20 mg of the sample was dispersed in a mixture of ultrapure water (500 μL), ethanol (480 μL), and Nafion solution (5 wt%, 20 μL). The mixture was ultrasonicated for 1h to achieve a uniform dispersion. Subsequently, 150 μL of the ink was drop-cast onto a nickel foam substrate (1 cm × 1 cm), resulting in a catalyst loading of ~3 mg cm^−2^. The electrode was then dried in air and used as the working electrode for electrochemical tests.

OER testing was carried out in 1M KOH aqueous solution. LSV curves were recorded in the potential range of 1.1–1.7 V (vs. RHE) at a scan rate of 5 mV s^−1^ with iR compensation applied. The electrochemical active surface area (ECSA) was estimated based on the electrochemical double-layer capacitance (C_dl_), which was determined by cyclic voltammetry (CV) at various scan rates (10, 20, 30, 40, 50 mV s^−1^) within the non-faradic region. C_dl_ was calculated by plotting half the difference between anodic and cathodic current density (j_a_−j_c_) at 1.04 V as a function of scan rate. CV tests for probing *OH adsorption were performed at a scan rate of 5 mV s^−1^ within a potential range of 0.5−1.1 V. In situ electrochemical impedance spectroscopy (EIS) measurements was performed over a frequency range of 100 kHz to 0.01 Hz, with applied potentials ranging from 1.30 to 1.50 V. All potentials were converted and reported versus the reversible hydrogen electrode (RHE).

The anion exchange membrane water electrolysis (AEMWE) single cell consists of an anion exchange membrane, electrodes, bipolar plates, current distributors, and end plates. The membrane electrode assembly (MEA) was fabricated using the catalyst-coated substrate (CCS) approach, wherein the anion exchange membrane is sandwiched between the cathode and anode. The anion exchange membrane (Sustainion X37-50 grade RT) was provided by Dioxide Materials. Co,Y-NiFeOOH and the commercial Pt/C (20 wt%) served as the anode and cathode catalysts, respectively. Catalyst ink preparation followed the same procedure as described for electrochemical experiments. AEMWE measurement was conducted using a Zahner electrochemical workstation (Zennium Pro, Germany). The single cell was operated at room temperature with 1 M KOH electrolyte supplied at a flow rate of 65 mL·min⁻^1^ to both electrodes.

***TOF value and Faradaic efficiency calculations:*** To assess intrinsic activity of the surface where the catalytic reaction actually occurs, TOF values were calculated using the following *Eqs.* (1−4):^[1]^

$\text{TOF=}\frac{\text{number of total oxygen turnovers / }\text{cm}^{\text{2}}\text{ of geometric area}}{\text{number of active sites / }\text{cm}^{\text{2}}\text{ of geometric area}}$ (1)

The total turnover number (TON) of oxygen was calculated based on the current density using Faraday’s law of electrolysis.

$\text{No. of }\text{O}_{\text{2}}\text{ = (}\text{j}\text{ }\frac{\text{mA}}{\text{cm}^{\text{2}}}\text{)(}\frac{\text{1 C}\text{s}^{\text{-1}}}{\text{1000 mA}}\text{)(}\frac{\text{1 mol of }\text{e}^{\text{-}}}{\text{96485.3 C}}\text{)(}\frac{\text{1 mol of }\text{O}_{\text{2}}}{\text{4 mol of }\text{e}^{\text{-}}}\text{)(}\frac{\text{6.022×}\text{10}^{\text{23}}\text{ }\text{O}_{\text{2}}\text{ moleculars}}{\text{1 mol }\text{O}_{\text{2}}}\text{)}$ (2)

Active sites per real surface area:

${\text{Active sites= (}\frac{\text{Atoms }_{\text{unit cell}}}{\text{V}_{\text{unit cell}}\text{ (}\text{nm}^{\text{3}}\text{)}}\text{)}}^{\frac{\text{2}}{\text{3}}}$ (3)

$\text{TOF=}\frac{\text{(}\text{1}\text{.}\text{56}\text{×}\text{10}^{\text{15}}\text{ }\frac{\text{O}_{\text{2}}\text{/s}}{\text{cm}^{\text{2}}}\text{ per }\frac{\text{mA}}{\text{cm}^{\text{2}}}\text{)}}{\text{surfacesites × }\text{A}_{\text{ECSA}}}$ | *j* | (4)

The Faradaic efficiency of the OER was determined by calculating the ratio of the experimentally measured volume of oxygen to the theoretically calculated volume, as described by *Eq.* (5).

$\text{Faradic efficiency=}\frac{\text{n}_{\text{a}}}{\text{n}_{\text{t}}}\text{=}\frac{\text{m}\text{×}\text{n}\text{×}\text{F}}{\text{I}\text{×}\text{t}}\text{×100 \%}$ (5)

$\text{m=}\frac{\text{V}}{\text{V}_{\text{m}}}$ (6)

Here, *m* represents the number of moles of O_2_ produced, *n* denotes the number of electrons transferred during the reaction, *F* is the Faraday constant, *I* is the applied current, *t* is the electrolysis time, *V* is the generated Oxygen volume, and *V*_m_ is molar volume of gas (24.5 L mol^−1^).

***In situ Raman spectroscopy*:** Raman spectra were collected using a Horiba−Jobin Yvon (LabRaman HR Evolution) Raman spectrometer equipped with a 532 nm excitation laser. Measurements were conducted in a custom-designed electrochemical cell with an Ag/AgCl reference electrode and a platinum wire counter electrode. The applied potential was swept from open-circuit potential (OCP) up to 1.50 V vs. RHE.

***In situ ATR-SEIRAS measurement*:** To elucidate the reaction mechanism, in situ attenuated total reflection surface-enhanced infrared absorption spectroscopy (ATR-SEIRAS) was employed to monitor reaction intermediates during the OER process. The measurements were carried out using a Bruker thermoelectric infrared spectrometer equipped with a liquid-nitrogen cooled Mercury−Cadmium−Telluride (MCT) detector. A customized spectroelectrochemical cell was assembled atop a Si prism for operando measurement. A platinum wire was used as the counter electrode while an Ag/AgCl served as the reference electrode. FTIR spectra were recorded by averaging 32 scans at a resolution of 8 cm^−1^ over a wavenumber range of 1000–4000 cm^−1^. Background spectra were collected at OCP in 1M KOH electrolyte. The applied potential was varied from OCP to 1.60 V vs. RHE.

***Theoretical simulation*:** Density functional theory (DFT) calculations were carried out by Vienna ab initio simulation package (VASP).^[2,3]^ The projector augmented-wave (PAW) method was employed to describe ion-electron interactions. The exchange–correlation functional was treated using the generalized gradient approximation (GGA) with the Perdew–Burke–Ernzerhof (PBE) parametrization. A plane-wave cutoff energy of 480 eV was applied, and van der Waals interactions were included using the semiempirical DFT-D3 correction method. Convergence thresholds were set to 10^−5^ eV for electronic energy and 0.02 eV Å^−1^ for atomic force. A *k*-point mesh of 4×1×1 was used for surface calculations. For *OH adsorption energy, the Co,Y-NiFeOOH slab model was constructed based on the (110) plane of NiFeOOH, with a vacuum layer of 15 Å along the c-axis to avoid interlayer interactions. To minimize interaction between adjacent adatoms, a sufficiently large supercell of 3×4 for NiFeOOH with (110) plane was employed.

Due to the limitations in achieving full coordination of metal sites in the model, the (110) plane of NiFeOOH was utilized to calculate the subsequent four-electron transfer steps of OER. The Gibbs free energy changes for the water oxidation steps, based on the dual-site synergistic mechanism (DSSM), were calculated according to *Eqs.* (7−11)^[4,5]^

OH^−^ + M_1_^*^ → M_1_−*OH + e^−^ (7)

OH^−^ + M_2_^*^ → M_2_−*OH + e^−^ (8)

M_1_−*OH + *OH^−^ → M_1_−O^*^ + e^−^ + H_2_O (9)

M_1_−O^*^ + M_2_−O^*^ → M_1_−O−O−M_2_ (10)

M_1_−O−O−M_2_ → M^*^ + O_2_ (11)

The intermediates adsorption energy E_ads_ for ^*^OH, ^*^O and *O−O* can be calculated using the ground-state energies from DFT.

ΔG_1_ = E_(*OH)_ – E_(*)_ − E_H2O_ + 1/2 E_H2_ + (ΔZPE − TΔS)_1_ – eU (12)

ΔG_2_ = E_(*O)_ – E_(*OH)_ + 1/2 E_H2_ + (ΔZPE − TΔS)_2_ −eU (13)

ΔG_3_ = E_(*O−O*)_ – E_(*O)_ – E_(*O)_ + (ΔZPE − TΔS)_3_ – eU (14)

ΔG_4_ = E_O2_ + E_(*)_ – E_(*O−O*)_ + (ΔZPE − TΔS)_4_ – eU (15)

The Gibbs free energy changes for the water oxidation steps using traditional adsorption evolution mechanism (AEM) were calculated using *Eqs.* (16−19):

M^*^ + OH^−^→ M−*OH + e^−^ (16)

M−*OH + OH^−^ → M−^*^O + e^−^ + H_2_O (17)

M−^*^O + OH^−^ → M−^*^OOH + e^−^ (18)

M−^*^OOH + OH^−^ → M^*^ + O_2_ + e^−^ + H_2_O (19)

The intermediates adsorption energies E_ads_ for M−^*^OH, M−^*^O and M−^*^OOH can be calculated using DFT ground state energies.

ΔG_1_ = E_(*OH)_ – E_(*)_ − E_H2O_ + 1/2 E_H2_ + (ΔZPE − TΔS)_1_ – eU (20)

ΔG_2_ = E_(*O)_ – E_(*OH)_ + 1/2 E_H2_ + (ΔZPE − TΔS)_2_ – eU (21)

ΔG_3_ = E_(*OOH)_ – E_(*O)_ − E_H2O_ + 1/2 E_H2_ + (ΔZPE − TΔS)_3_ − eU (22)

ΔG_4_ = E_O2_ + E_(*)_ – E_(*OOH)_ + 1/2 E_H2_ + (ΔZPE − TΔS)_4_ − eU (23)

**2. Figures and tables**

**Table S1.** Molar ratios of elements in Ni_x_Fe_y_OOH measured by ICP-OES.

| **Samples** | **Ni** | **Fe** |
| --- | --- | --- |
| Ni_9_Fe_1_OOH | 0.89 | 0.11 |
| Ni_8_Fe_2_OOH | 0.81 | 0.19 |
| Ni_7_Fe_3_OOH | 0.68 | 0.32 |


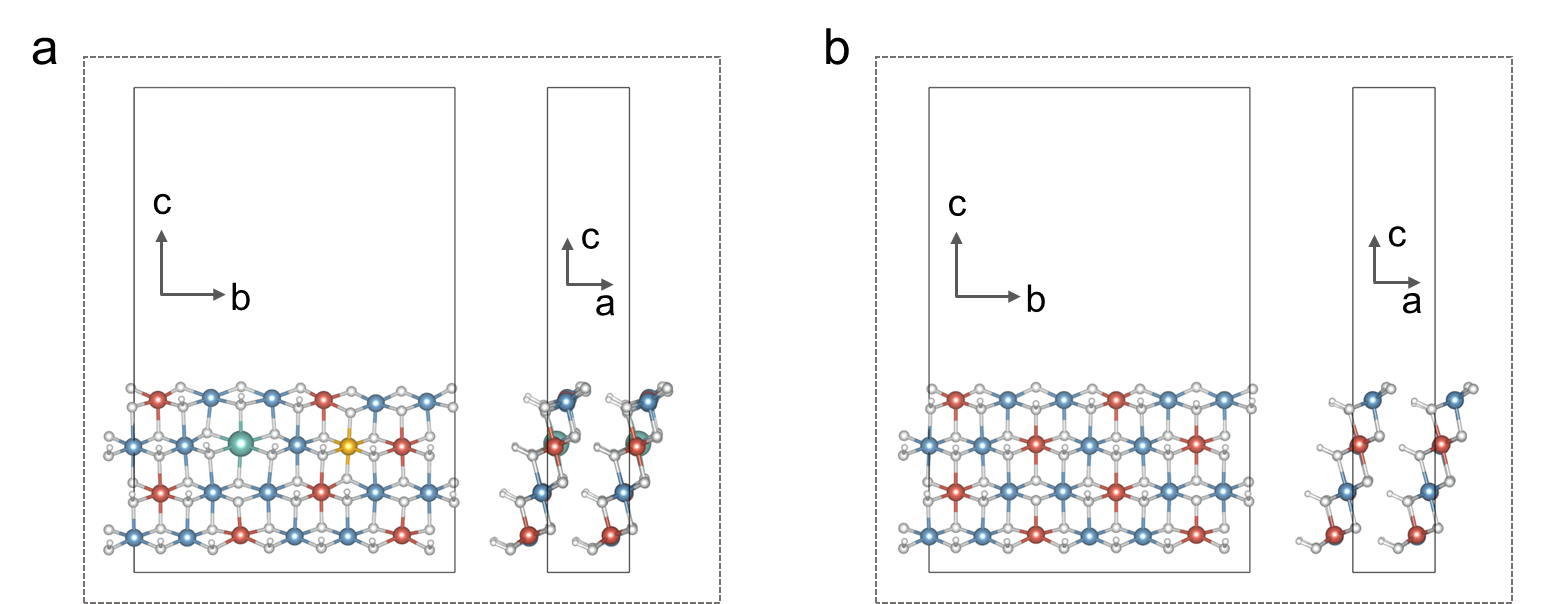


**Figure S1.** Atomic structures of (a) Co,Y-NiFeOOH and (b) NiFeOOH viewed from side and top views. The blue, red, yellow, green, gray, and white spheres represent Ni, Fe, Co, Y, O, and H, respectively.


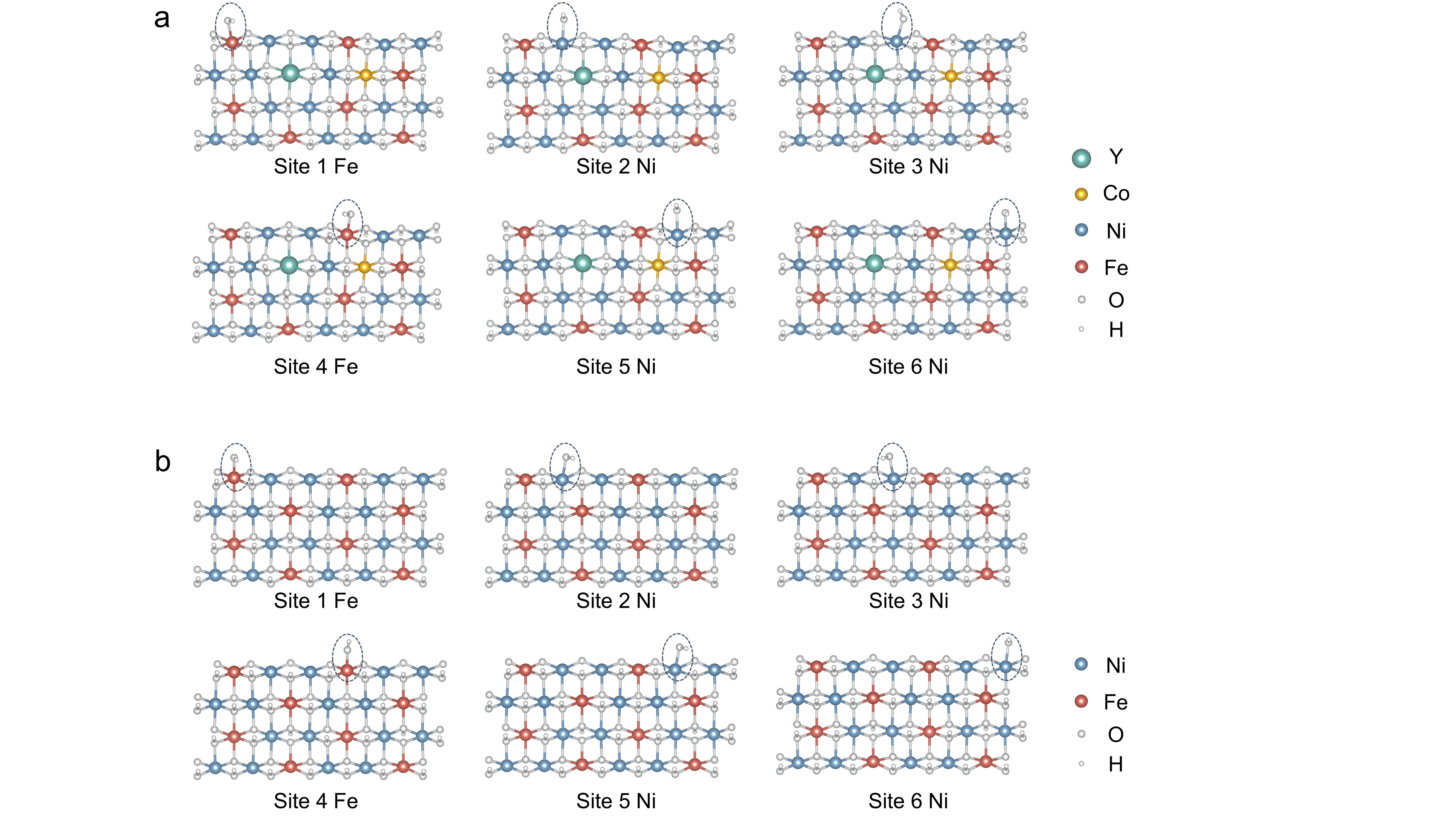


**Figure S2.** Atomic structures with *OH adsorbed at different sites on the (110) plane of (a) Co,Y-NiFeOOH and (b) NiFeOOH.

The *OH adsorption energy was calculated using the following equation, E_ads_ = E_total_ − E_slab_ − E_mol_, where E_total_ is the total energy for the adsorption state, E_slab_ is the energy of pure surface, and E_mol_ is the energy of adsorbed molecule.

**Figure S3.** XRD pattern of Co,Y-NiFeOOH.

No distinct diffraction peaks are observed in the XRD pattern, demonstrating the amorphous structure of Co,Y-NiFeOOH.^[6]^

**Table S2.** The molar ratios of elements in Co,Y-NiFeOOH and NiFeOOH were measured by ICP-OES.

| **Samples** | **Ni** | **Fe** | **Co** | **Y** |
| --- | --- | --- | --- | --- |
| Co,Y-NiFeOOH | 0.68 | 0.19 | 0.07 | 0.06 |
| NiFeOOH | 0.76 | 0.24 | / | / |

The molar ratios of elements were further quantified by ICP-OES, yielding a Ni:Fe:Co:Y molar ratio of approximately 7:2:0.5:0.5 for Co,Y-NiFeOOH, compared to a Ni:Fe ratio of 7:2 for undoped NiFeOOH.

**
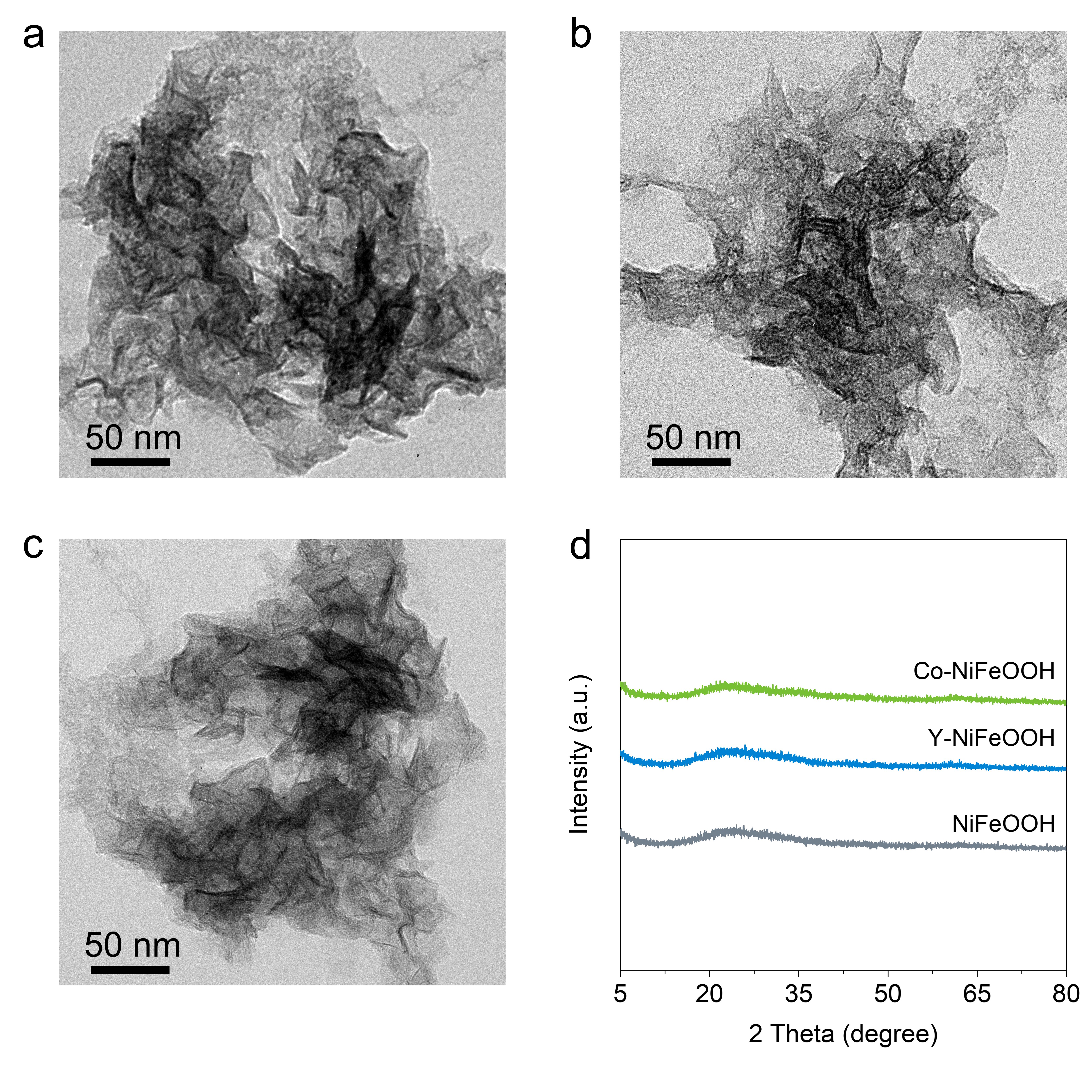
**

**Figure S4.** Characterizations on control samples. TEM images of (a) Co-NiFeOOH, (b) Y-NiFeOOH and (c) NiFeOOH. (d) XRD patterns of Co-NiFeOOH, Y-NiFeOOH and NiFeOOH.

The TEM observations reveal the presence of nanoscale lamellar morphology analogous to that of Co,Y-NiFeOOH, and their XRD patterns exhibit distinct amorphous characteristics.


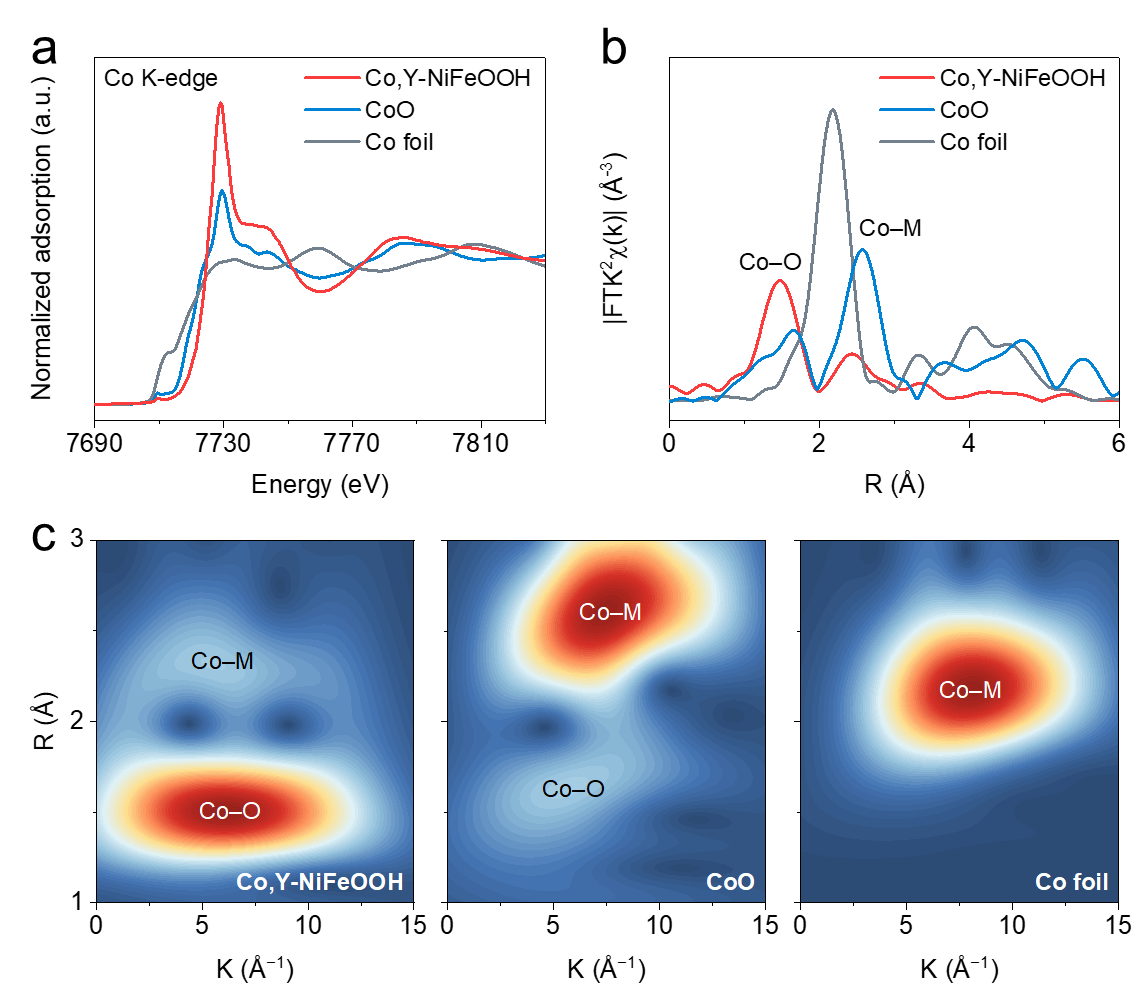


**Figure S5.** (a) Co K-edge XANES spectra with magnified marked region inserted. (b) Fourier-transform spectra from EXAFS at Co K-edge. (c) Corresponding WT-EXAFS plots. CoO and Co foil were used for reference.

The Co K-edge XANES spectra reveal that the absorption edge of Co in Co,Y-doped NiFeOOH shifts to the right relative to CoO, suggesting that the valence state of Co exceeds +2. These results demonstrate that Co,Y-doped NiFeOOH possesses distinct coordination environments for Co–O and Co–M (second-shell scattering of Co–O–M). Furthermore, wavelet-transform (WT)-EXAFS analysis confirms the coordination environments of Co–O and Co–M in Co,Y-doped NiFeOOH.^[7]^

**Table S3.** Structural parameters extracted from EXAFS fitting.

| Samples | Path | CN | R(Å) | σ^2^(10^−3^Å^2^) | ΔE_0_(eV) | R factor |
| --- | --- | --- | --- | --- | --- | --- |
| Ni foil | Ni–Ni | 12* | 2.48 | 5.7 | 7.0 | 0.002 |
| Fe foil | Fe–Fe | 12* | 2.47 | 4.2 | 4.8 | 0.003 |
| Co foil | Co–Co | 12* | 2.48 | 5.2 | 1.1 | 0.005 |
| Co,Y-NiFeOOH-XANES | Ni–O | 5.6 | 2.07 | 6.2 | 1.0 | 0.009 |
|  | Ni–M | 4.6 | 3.09 | 7.9 |  |  |
|  | Fe–O | 5.1 | 1.97 | 6.9 | 1.5 | 0.010 |
|  | Fe–M | 5.5 | 3.02 | 7.8 |  |  |
|  | Co–O | 5.3 | 1.93 | 7.6 | 1.1 | 0.015 |
|  | Co–M | 5.2 | 2.95 | 8.2 |  |  |
| NiFeOOH-XANES | Ni–O | 5.6 | 2.06 | 5.5 | 2.2 | 0.008 |
|  | Ni–M | 4.7 | 3.07 | 7.1 |  |  |
|  | Fe–O | 5.1 | 1.97 | 5.3 | 1.8 | 0.011 |
|  | Fe–M | 5.5 | 3.01 | 6.8 |  |  |

S_0_^2^ is the amplitude reduction factor; CN is the coordination number; R is interatomic distance (the bond length between Ru central atoms and surrounding coordination atoms); σ^2^ is Debye-Waller factor (a measure of thermal and static disorder in absorber-scatterer distances); ΔE_0_ is edge-energy shift (the difference between the zero kinetic energy value of the sample and that of the theoretical model). R factor is used to value the goodness of the fitting.

* This value was fixed during EXAFS fitting, based on the known structure.

Error bounds that characterize the structural parameters obtained by EXAFS spectroscopy were estimated as N ± 20 %; R ± 1 %; σ^2^ ± 20 %; ΔE_0_ ± 20 %.

**
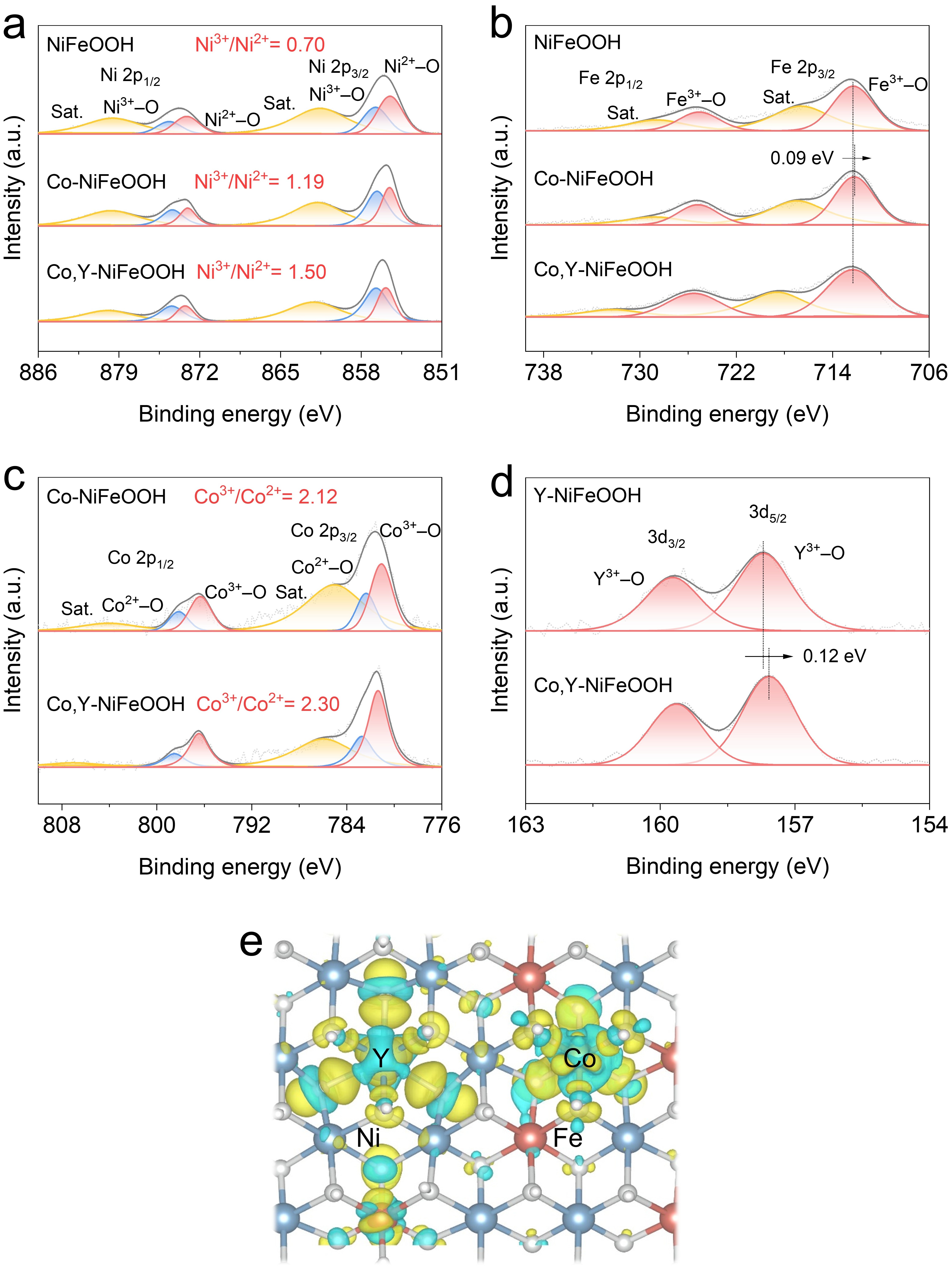
**

**Figure S6.** High-resolution XPS spectra of (a) Ni 2p, (b) Fe 2p, (c) Co 2p, and (d) Y 3d. (e) Differential charge density plot of Y and Co sites in Co,Y-NiFeOOH. Yellow and cyan represent charge accumulation and consumption, respectively.

To investigate the electronic microenvironment modulation induced by Co and Y doping on, a combination of XPS analysis and computational simulations was utilized. As shown in Figure S6a, the peaks at 855.9 and 873.3 eV correspond to Ni 2p_3/2_ and Ni 2p_1/2_ of Ni^2+^–O respectively, while the peaks at 856.9 and 874.3 eV are attributed to Ni 2p_3/2_ and Ni 2p_1/2_ of Ni^3+^–O.^[8]^ Notably, Co doping elevates the oxidation state of Ni, an effect further enhanced by Y co-doping. This synergistic increase in Ni oxidation state enhances the Lewis acidity of Ni sites, strengthening their hydroxyl adsorption capacity. For the Fe 2p XPS spectra (Figure S6b), the peaks at 712.3 eV and 725.6 eV are assigned to Fe 2p_3/2_ and Fe 2p_1/2_ of Fe^3+^–O respectively.^[9]^ While Co,Y co-doping remains the Fe valence unchanged, the elongation of Fe–O–M bonds occur due to the large ionic radius of Y, potentially modulating hydroxyl adsorption at Fe sites. In addition, the XPS results further reveal the electron interactions between the doped Y and Co atoms (Figures S6c, S6d). Specifically, Y doping increased the oxidation of Co, and Co doping decreased the oxidation of Y, indicating electron transfer from Co to Y.

To elucidate charge redistribution, we computed the differential charge density at the Co and Y sites in Co,Y-doped NiFeOOH (Figure S6e). The electron accumulation state around Co and Y confirms their strong electron-withdrawing character, consistent with charge transfer from Ni to the dopants. This systematic electron redistribution demonstrates the synergistic impact of Co and Y doping on the electronic structure and catalytic performance of NiFeOOH.

**Figure S7.** LSV curves without iR correction.

**Table S4.** Comparison of our work with recently reported OER catalysts in 1M KOH.

| **Catalysts** | **η/mV**  **@ 1 A cm**^−^**^2^** | **Tafel slope**  **(mV dec**^−^**^1^)** | **Durability**  **(Conservation rate)** | **Reference** |
| --- | --- | --- | --- | --- |
| Co,Y-NiFeOOH | 270 | 30.7 | 1500 h @ 1000 mA cm^−2^, 98 % | This work |
| *V*_Cr_,Co-NiFeOOH | 300 | 39.5 | 500 h @ 500 mA cm^−2^, 98 % | J. Am. Chem. Soc., 2025^[6]^ |
| NiFe(S)/NM | 370 | 41.4 | 500 h @ 300 mA cm^−2^, 99 % | Angew. Chem. Int. Ed., 2025^[10]^ |
| NiFe-LDH/Ni_3_S_2_ | 300 | 45.1 | 220 h @ 800 mA cm^−2^, 99 % | Angew. Chem. Int. Ed., 2025^[11]^ |
| FeMoOOH | 340 | 72.3 | 1000 h @ 1500 mA cm^−2^, 94 % | Energy Environ. Sci., 2025^[12]^ |
| NiFe-TCPP | 290 | 41.8 | 1000 h @ 500 mA cm^−2^, 99 % | Adv. Funct. Mater., 2025^[13]^ |
| CrO_4_^2–^-NiFe LDH/Cr_2_O_3_ | 355 | 37.2 | 1000 h @ 1000 mA cm^−2^, 99 % | Nat. Commun., 2024^[14]^ |
| FeNiHOF | 280 | 34.8 | 1000 h @ 1000 mA cm^−2^, 99 % | Nat. Commun., 2024^[15]^ |
| Nei-Ir_1_/CoGaOOH | 320 | 47.0 | 700 h @ 100 mA cm^−2^, 99 % | Angew. Chem. Int. Ed., 2024^[16]^ |
| (NiFe)C_2_O_4_ | 328 | 39.9 | 600 h @ 1000 mA cm^−2^, 97 % | Angew. Chem. Int. Ed., 2024^[17]^ |
| FeOOH/Co(OH)_2_ | 304 | 38.0 | 150 h @ 500 mA cm^−2^, 98 % | Adv. Mater., 2024^[18]^ |
| CoFeOOH/CeO_2−x_N_x_ | 297 | 33.0 | 1000 h @ 1900 mA cm^−2^, 92 % | Nat. Commun., 2023^[19]^ |
| Fe-NiCo-LDH | 400 | 51.9 | 500 h @ 500 mA cm^−2^, 100 % | Adv. Funct. Mater., 2023^[20]^ |

**Figure S8.** Mass activity curves of all samples (normalized by catalyst loading).

It reveals that our optimized catalyst achieves 332 mA mg⁻^1^ at 270 mV overpotential (corresponding to 1.5 V potential), which is ~9.76, 11.86, and 27.67 times that of Co-NiFeOOH (34 mA mg⁻^1^), Y-NiFeOOH (28 mA mg⁻^1^), and NiFeOOH (12 mA mg⁻^1^), respectively.

**Figure S9.** TOF-value plot of Co,Y-NiFeOOH and its control samples at 270 mV overpotential.

The TOF values based on the number of Ni and Fe atoms at 270 mV were used to present the transfer number of O_2_ molecules per surface active site per unit time. Co,Y-NiFeOOH has a TOF value of 7.07 s^−1^, which is ~6.73, 6.61, and 18.13 times that of Co-NiFeOOH (1.05 s^−1^), Y-NiFeOOH (1.07 s^−1^), and NiFeOOH (0.39 s^−1^), respectively.


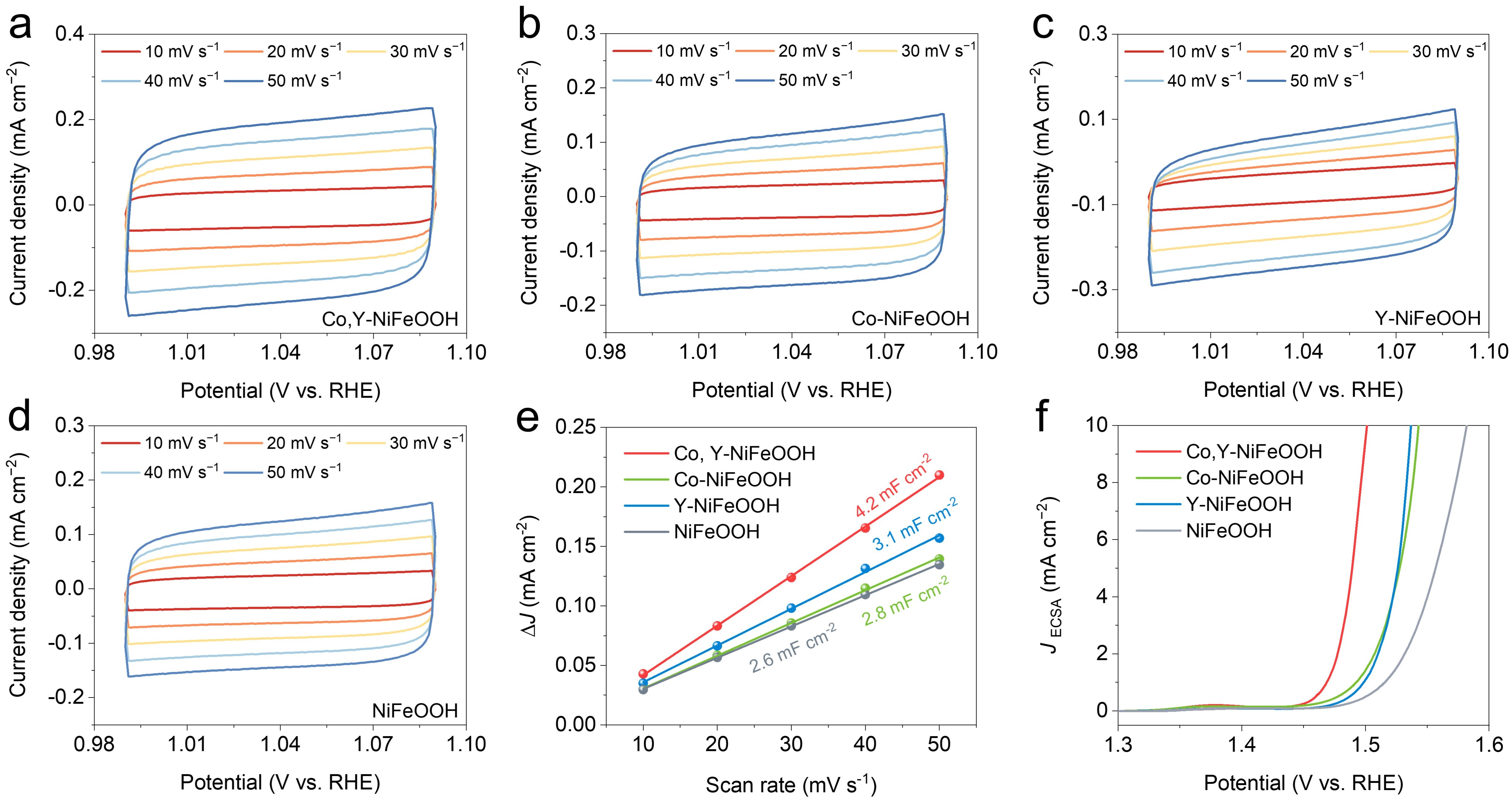


**Figure S10.** CV curves of (a) Co,Y-NiFeOOH, (b) Co-NiFeOOH, (c) Y-NiFeOOH and (d) NiFeOOH at different scan rates (10, 20, 30, 40 and 50 mV s^−1^) tested in the non-Faradaic potential region. (e) C_dl_ values of the above electrodes calculated from the CV results. (f) ECSA normalized LSV curves.

The LSV curves were normalized to the electrochemical surface area (ECSA). Co,Y-NiFeOOH exhibits a high specific activity of 9.8 mA cm^−2^_ECSA_ at an overpotential of 270 mV, which is 5.6, 7.4, and 16.8 times greater than that of Co-NiFeOOH (1.4 mA cm^−2^_ECSA_), Y-NiFeOOH (1.1 mA cm^−2^_ECSA_) and NiFeOOH (0.5 mA cm^−2^_ECSA_), respectively.

**Figure S11.** Nyquist plots with simulated circuit and comparison of R_ct_ values inserted.

The EIS analysis reveals that the charge transfer resistance (R_ct_) of Co,Y-NiFeOOH is 0.86 Ω, lower than those of Co-NiFeOOH (3.60 Ω), Y-NiFeOOH (2.86 Ω), and NiFeOOH (6.98 Ω).


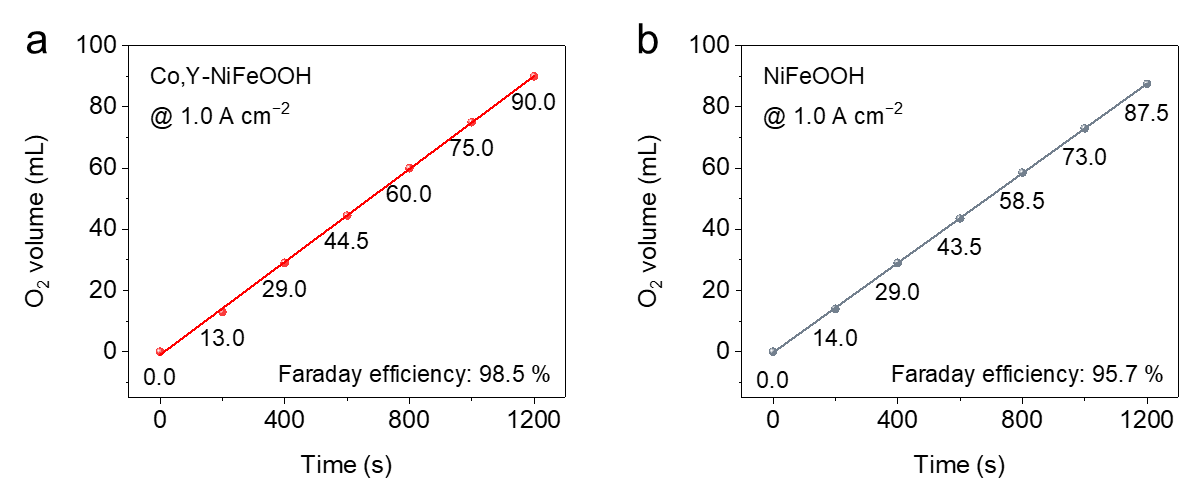


**Figure S12.** Experimentally measured O_2_ volume and Faraday efficiency of (a) Co,Y-NiFeOOH and (b) NiFeOOH under 1.0 A cm^−2^ current densities.

Faraday efficiency tests were conducted using the drainage method in an H-type electrolytic cell, which was separated by an anion exchange membrane. Co,Y-NiFeOOH exhibits Faraday efficiency of 98.5 % at current density of 1.0 A cm^−2^, surpassing Faraday efficiency of NiFeOOH (95.7 %).

**Figure S13.** Five unstable types of rectangular waves employed as signal input to simulate fluctuating state with the range from 20 to 120 % based on fixed current density of 1 A cm^−2^.


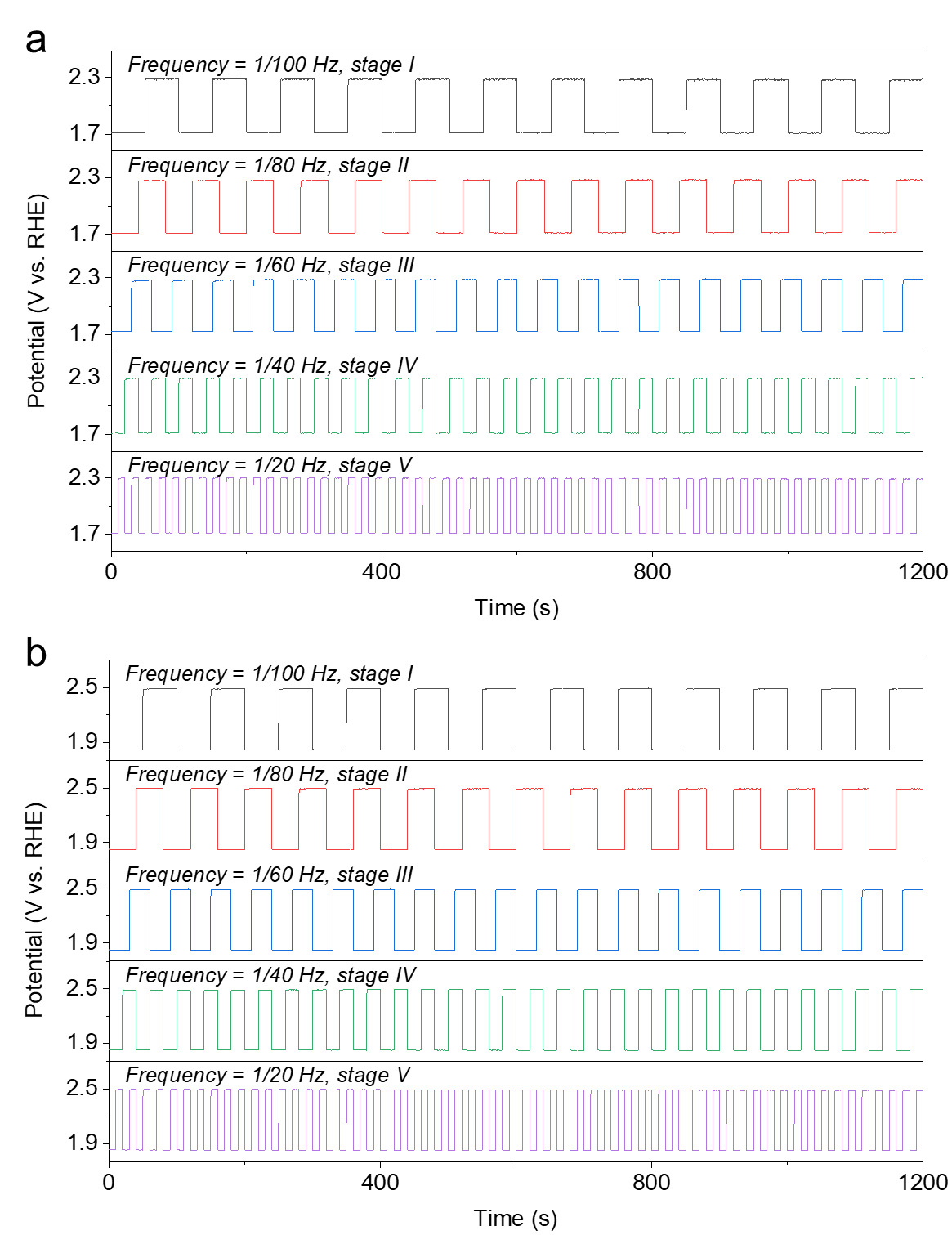


**Figure S14.** Measured origin data of fluctuation state tests. (a) Co,Y-NiFeOOH and (b) NiFeOOH.

**Table S5.** Generated Oxygen volume and its Faraday efficiency for fluctuation state tests on Co,Y-NiFeOOH and NiFeOOH.

| Samples |  | Stage *I* | Stage *II* | Stage *III* | Stage *IV* | Stage *V* |
| --- | --- | --- | --- | --- | --- | --- |
| Co,Y-NiFeOOH | O_2_ volume (mL) | 52.6 | 52.4 | 52.1 | 51.9 | 51.7 |
|  | FE (%) | 98.6 | 98.3 | 97.7 | 97.3 | 97.0 |
| NiFeOOH | O_2_ volume (mL) | 51.3 | 50.9 | 50.2 | 49.6 | 49.2 |
|  | FE (%) | 96.2 | 95.5 | 94.1 | 93.0 | 92.3 |

The Faradaic efficiency was calculated using Equations 5 and 6 in the “TOF value and Faradaic efficiency calculations” section. Specifically, Equation 6 was used to determine the number of moles of O_2_ produced. The total applied duration was 600 s at a current density of 0.2 A cm⁻^2^, and also 600 s at a current density of 1.2 A cm⁻^2^. Therefore, the total electric charge (I×t) is calculated as 0.2×600 + 1.2×600 = 840. In Equations 5, *n* (the number of electrons transferred per mole of product) is 4, and *F* (Faraday constant) is 96485 C mol⁻^1^.


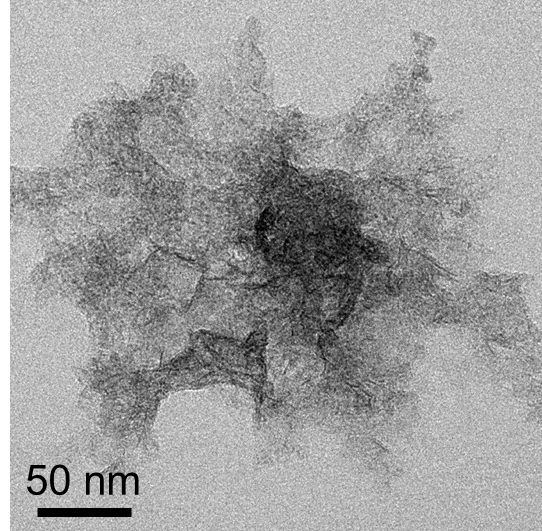


**Figure S15.** TEM image of Co,Y-NiFeOOH after stability testing, exhibiting sheet-like structure with a little aggregation.

**Table S6.** Molar ratios of elements in Co,Y-NiFeOOH measured by ICP-OES before and after stability testing.

| **Co,Y-NiFeOOH** | **Ni** | **Fe** | **Co** | **Y** |
| --- | --- | --- | --- | --- |
| Before | 0.68 | 0.19 | 0.07 | 0.06 |
| After | 0.71 | 0.17 | 0.06 | 0.06 |


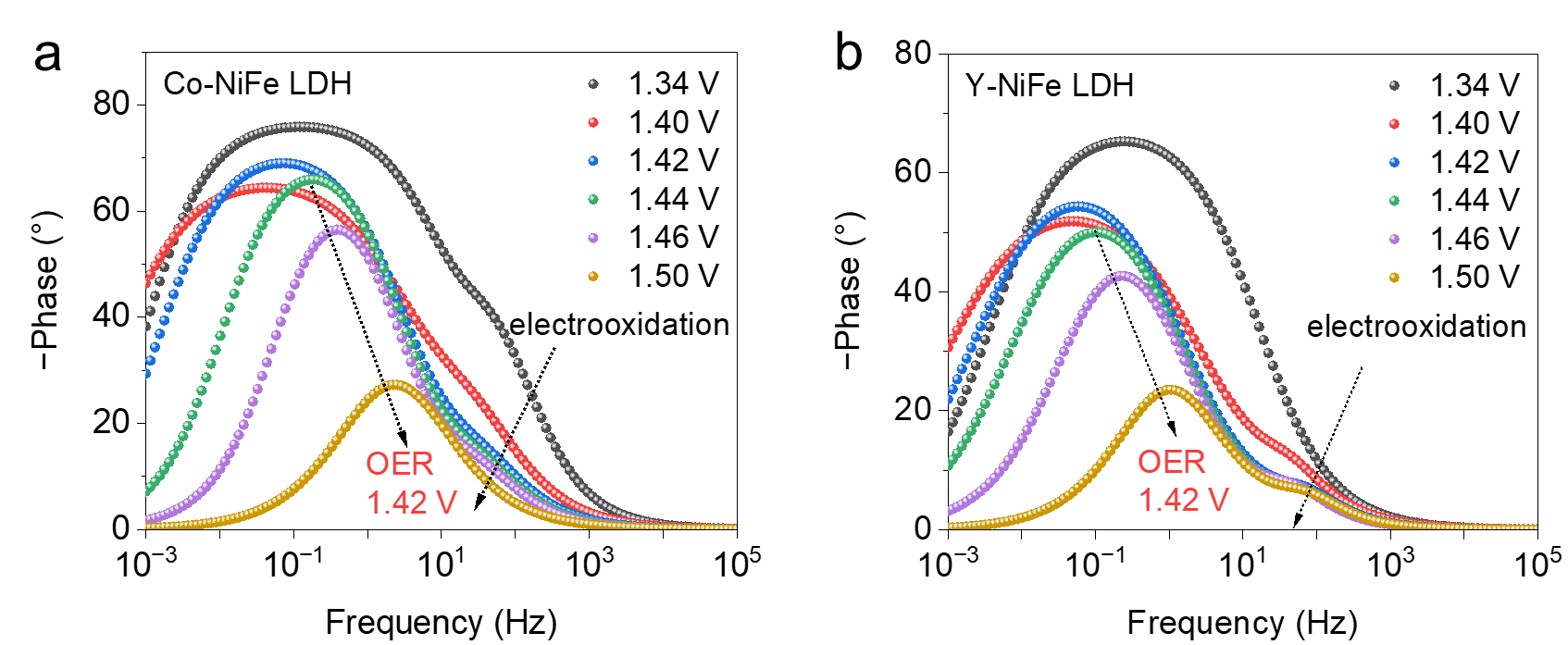


**Figure S16.** In situ electrochemical process corresponding Bode plots of (a) Co-NiFe LDH and (b) Y-NiFe LDH at different potentials from 1.34 to 1.50 V.

In situ EIS measurements were utilized to assess the initial OER potentials of catalytic materials. In Bode diagrams, the high-frequency regions correspond to the electrooxidation of catalytic materials, while the low-frequency regions are associated with the OER process.^[21]^ The highest phase angle in the low-frequency region corresponds to the initial OER potential.^[22]^ During the OER process, shoulder peaks shift toward higher frequencies and lower phase angles as the potential increases further, which is attributed to redox reactions at the electrode-electrolyte interface.^[23]^ Among all analyzed samples, Co,Y-NiFe LDH demonstrates rapidly diminishing peaks in the high-frequency region, an initial OER potential of 1.40 V (the lowest among all tested samples), and a faster change in phase angle, indicating that Co,Y-NiFeOOH (activated Co,Y-NiFe LDH) exhibits superior reaction kinetics compared to its three counterparts. Both Co-NiFe LDH and Y-NiFe LDH show an initial OER potential of 1.42 V, which lies between that of Co,Y-NiFe LDH (1.40 V) and NiFeOOH (1.44 V).


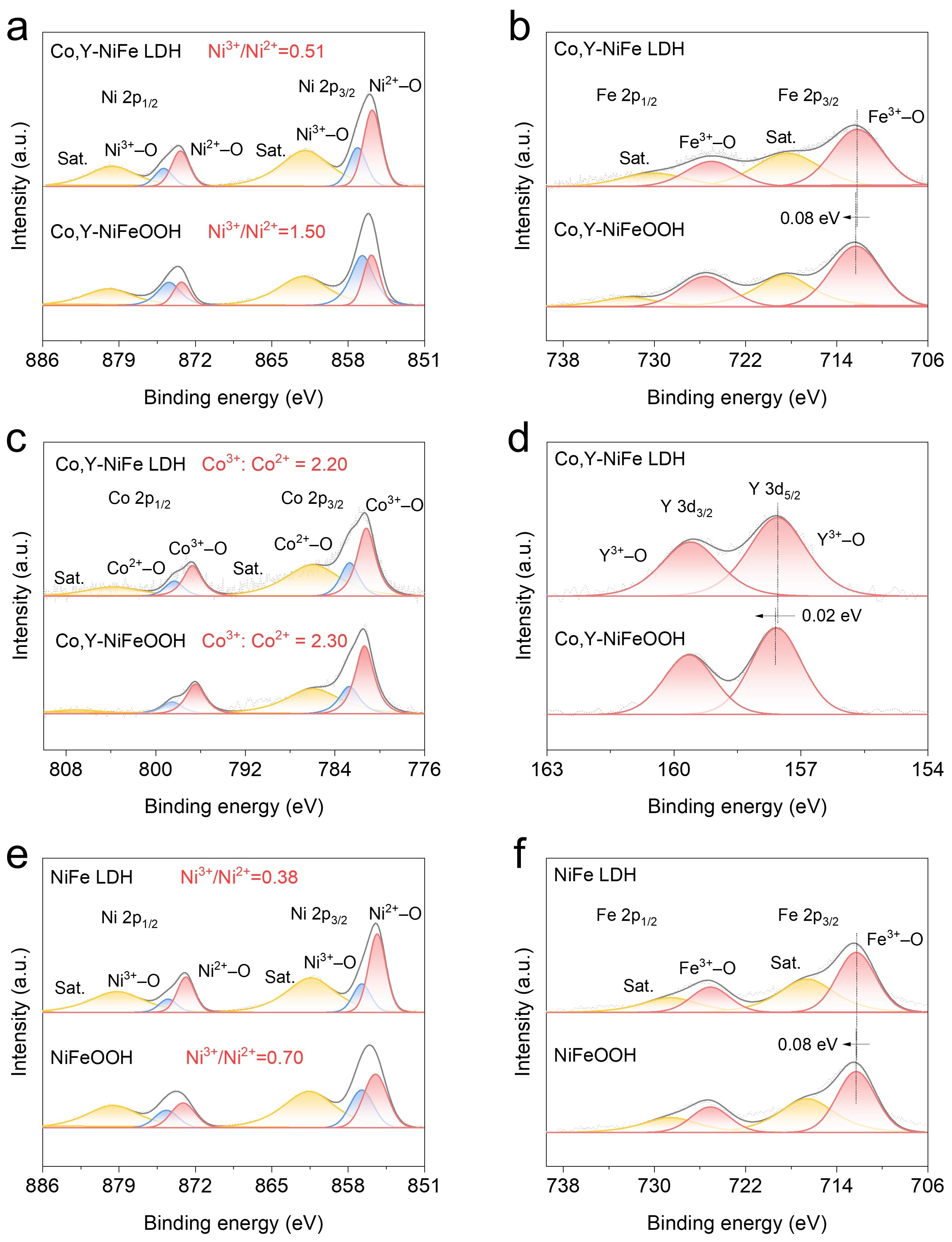


**Figure S17.** High-resolution XPS spectra of (a) Ni 2p, (b) Fe 2p, (c) Co 2p, and (d) Y 3d for Co,Y-NiFe LDH and Co,Y-NiFeOOH. High-resolution XPS spectra of (e) Ni 2p, and (f) Fe 2p for NiFe LDH and NiFeOOH.

XPS analysis was employed to investigate the valence state evolution of Ni, Co, Fe, and Y during the OER activation process (LDH→OOH transformation, LDH corresponding to OCP). As shown in Figure S17a, the Ni 2p spectra display characteristic peaks for both Ni^2+^ (red peaks: Ni 2p_3/2_ and Ni 2p_1/2_ of Ni^2+^–O) and Ni^3+^ (blue peaks: Ni 2p_3/2_ and Ni 2p_1/2_ of Ni^3+^–O). Notably, the Ni^3+^/Ni^2+^ ratio increases significantly from 0.51 in the LDH phase to 1.50 in the OOH phase for Co,Y-NiFe samples, indicating pronounced Ni oxidation during activation. The Fe 2p spectra (Figure S17b) reveal that iron maintains its Fe^3+^ state (red peaks: Fe 2p_3/2_ and Fe 2p_1/2_ of Fe^3+^–O) with minimal binding energy shifts (~0.08 eV). Additionally, XPS analysis of Co and Y (Figures S17c and S17d) shows an increased Co^3+^/Co^2+^ ratio from 2.20 to 2.30, and Y^3+^ states with a shift toward higher binding energy, demonstrating the elevated oxidation states. For comparison, undoped NiFe samples show a less increase in the Ni^3+^/Ni^2+^ ratio (from 0.38 to 0.70, Figure S17e) during activation. The difference clearly demonstrates the catalytic enhancement effect of Co,Y co-doping, which accelerates the oxidation kinetics of Ni centers. For Fe 2p spectra (Figure S17f), the behavior is consistent in both doped and undoped samples.


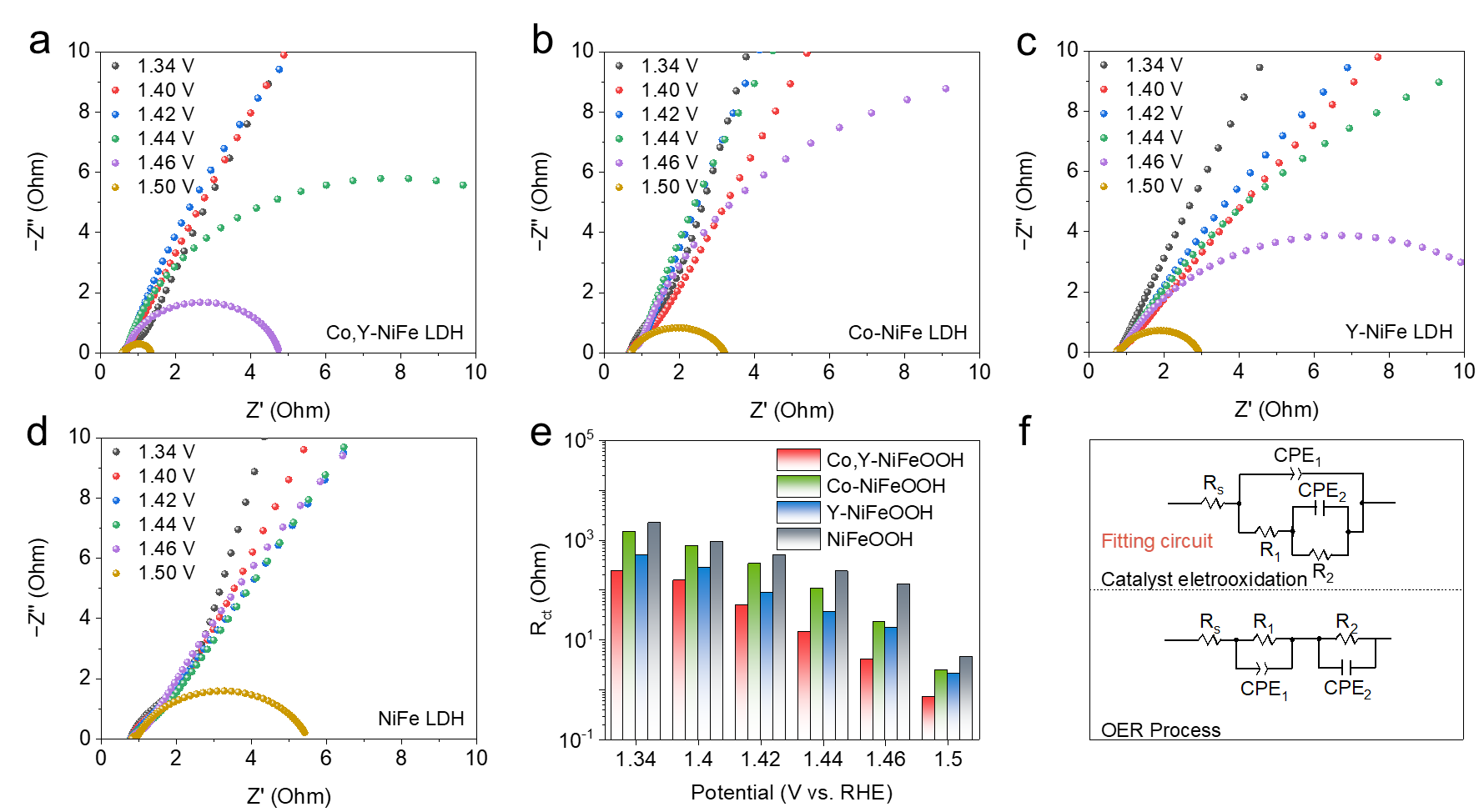


**Figure S18.** In situ electrochemical process corresponding Nyquist plots of (a) Co,Y-NiFe LDH, (b) Co-NiFe LDH, (c) Y-NiFe LDH, and (d) NiFe LDH at different potentials from 1.34 to 1.50 V vs RHE. (e) Corresponding R_ct_ values at different potentials. (f) Fitted circuits for *OH adsorption process and OER process.

The R_ct_ values of Co,Y-NiFe LDH and its three control samples were summarized, encompassing both the electrooxidation and OER processes.^[24]^ The R_ct_ values for Co,Y-NiFe LDH are consistently lower than those of the other materials across all potentials, indicating that the synergistic effect of Co and Y doping enhances the catalytic activity of Ni and Fe, promotes electron transfer, and consequently accelerates the OER kinetics.


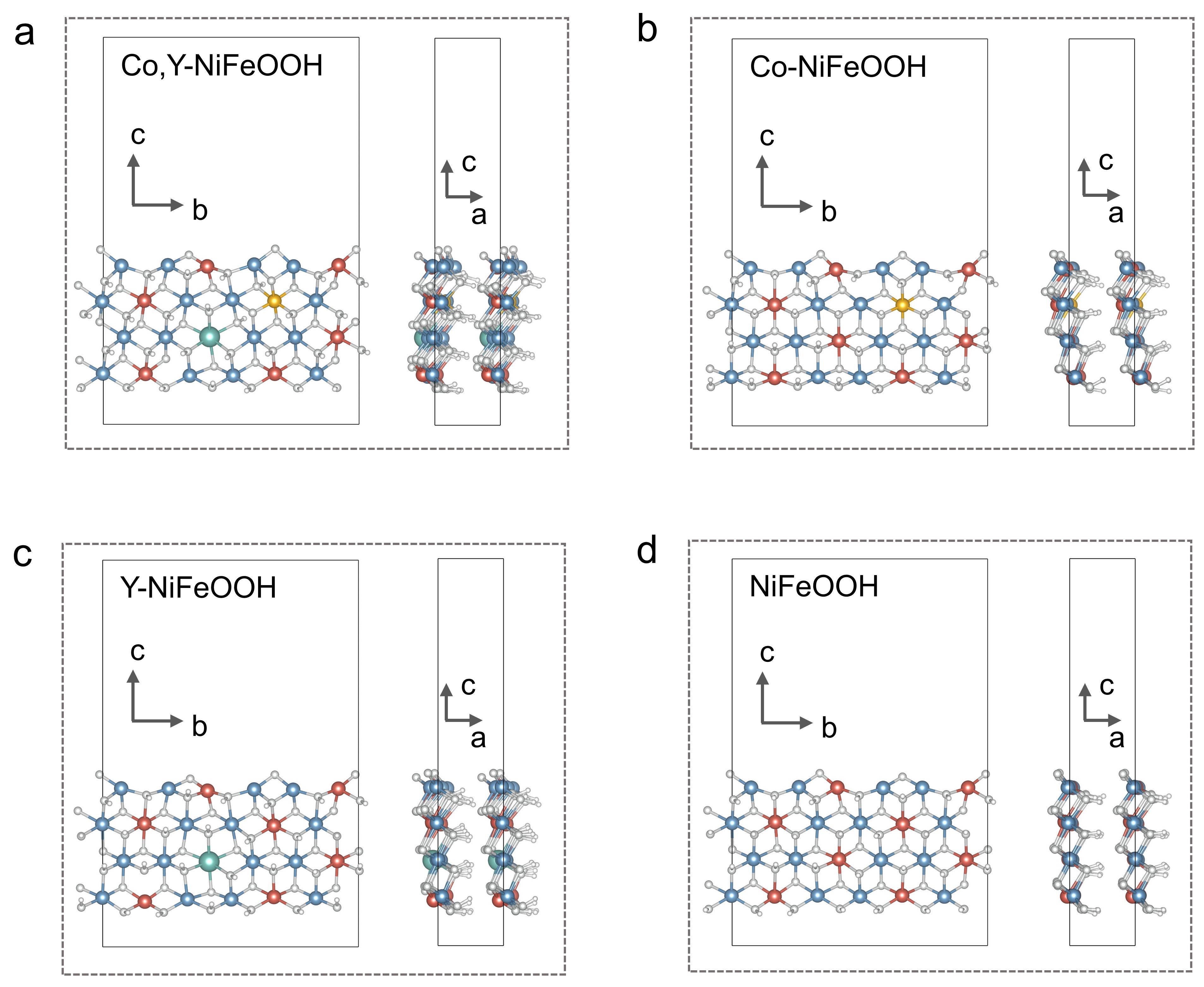


**Figure S19.** Atomic structures of (a) Co,Y-NiFeOOH, (b) Co-NiFeOOH, (c) Y-NiFeOOH and (b) NiFeOOH as viewed from different perspectives. The blue, red, yellow, green, gray, and white spheres represent Ni, Fe, Co, Y, O, and H, respectively.


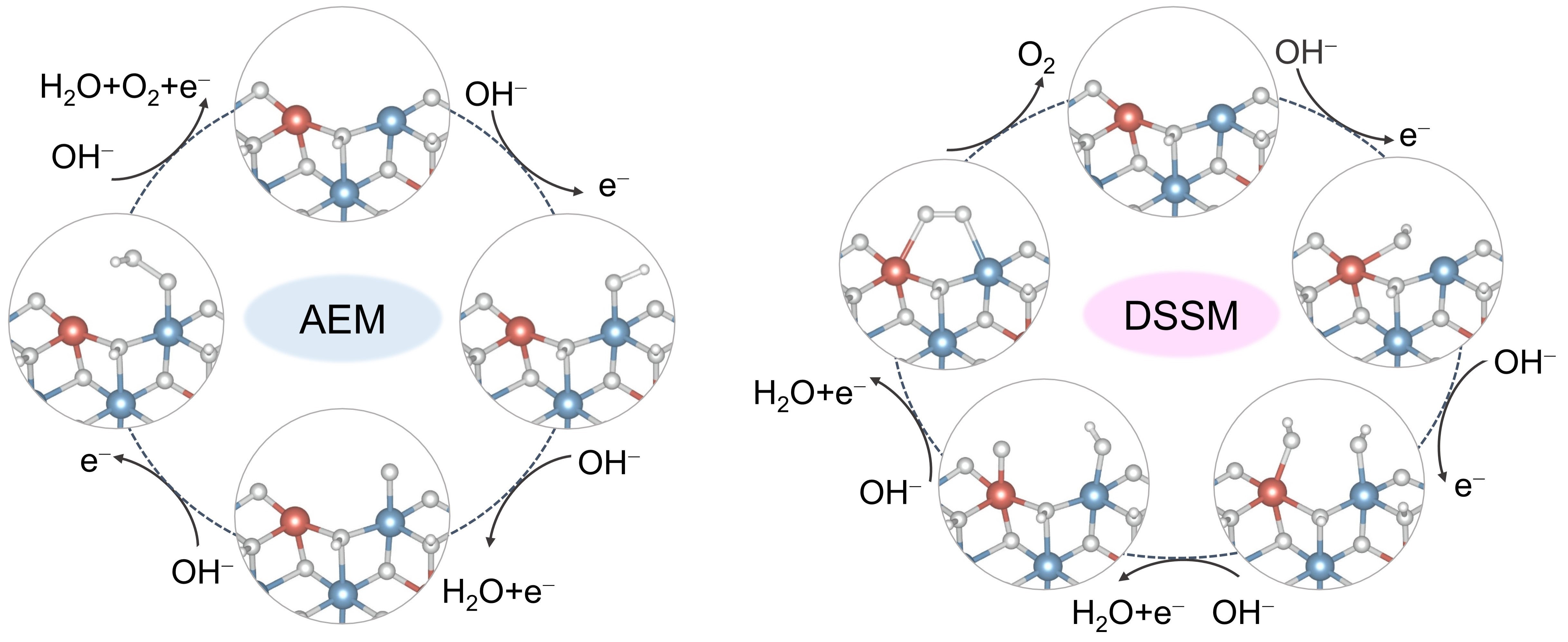


**Figure S20.** Atomic structures of reaction intermediates of AEM and DSSM pathways for NiFeOOH during OER process.


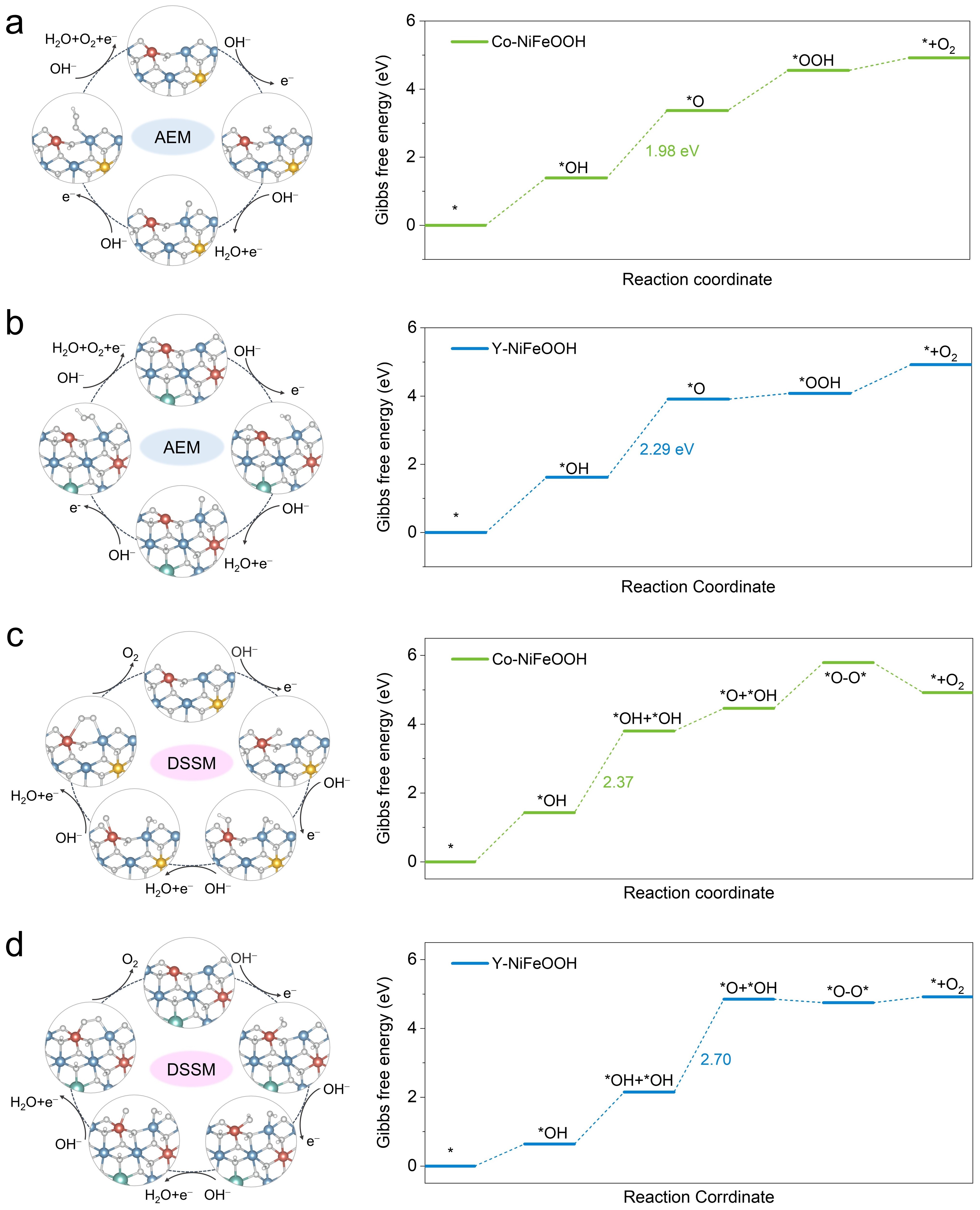


**Figure S21.** Atomic structures and corresponding Gibbs free energy diagrams for (a) AEM pathway of Co-NiFeOOH, (b) AEM pathway of Y-NiFeOOH, (c) DSSM pathway of Co-NiFeOOH and (d) DSSM pathway of Y-NiFeOOH.

**Table S7.** Comparison of our work with recently reported OER catalysts for AEMWE in 1M KOH.

| **Cathode Catalysts** | **Anode Catalysts** | **Temperature**  **(°C)** | **Area**  **(cm^2^)** | **Voltage (V)** | **Reference** |
| --- | --- | --- | --- | --- | --- |
| Pt/C | Co,Y-NiFeOOH | 25 | 25 | 1.94 @ 500 mA cm^−2^  2.13 @ 1000 mA cm^−2^ | This work |
| Pt/C | Co,Y-NiFeOOH | 25 | 1 | 1.74 @ 500 mA cm^−2^  1.80 @ 1000 mA cm^−2^ | This work |
| Pt/C | Ni/Co-TAPP-HNA/rGO | 25 | 1 | 2.09 @ 500 mA cm^−2^  2.45 @ 1000 mA cm^−2^ | Adv. Energy Mater., 2025^[25]^ |
| Pt/C | LC-NiMn-LMF | 25 | 1 | 1.87 @ 500 mA cm^−2^ | Adv. Funct. Mater., 2025^[26]^ |
| Pt/C | *A-*Fe_2_S_1_N_5_/SNC | 25 | 1 | 1.94 @ 500 mA cm^−2^  2.18 @ 1000 mA cm^−2^ | Nat. Commun., 2024^[27]^ |
| CoN/VN@NF | P-CoVO@NF | 25 | 2.25 | 1.96 @ 500 mA cm^−2^ | Adv. Mater., 2024^[21]^ |
| NiCoP_v_@NF | NiCoP_v_@NF | 25 | 1 | 1.93 @ 500 mA cm^−2^  2.01 @ 1000 mA cm^−2^ | Adv. Energy Mater., 2024^[28]^ |
| Pt QDs@Ni_3_N-MoN/Ti | Pt QDs@Ni_3_N-MoN/Ti | 25 | 1 | 1.95 @ 500 mA cm^−2^  2.18 @ 1000 mA cm^−2^ | Adv. Funct. Mater., 2024^[29]^ |
| Pt_1_/Co(OH)_2_ | Ir_1_/Co(OH)_2_ | 75 | 1 | 2.15 @ 500 mA cm^−2^ | Angew. Chem. Int. Ed., 2023^[30]^ |
| Pt@S-NiFe LDHs | S-NiFe LDHs | 25 | 1 | 2.50 @ 500 mA cm^−2^ | Adv. Mater., 2023^[31]^ |
| Pt mesh | Zn, S-Fe_3_O_4_-FeOOH/IF | 25 | 1 | 1.80 @500 mA cm^−2^  2.00 @1000 mA cm^−2^ | Adv. Funct. Mater., 2023^[32]^ |

**Figure S22.** Five unstable types of rectangular waves employed as signal input to simulate fluctuating state with the range from 20 to 120 % based on the fixed current density of 0.5 A cm^−2^, tested in AEMWE with the electrode area of 25 cm^2^.

**Figure S23.** Measured origin data of fluctuation state for Co,Y-NiFeOOH tested in AEMWE with the electrode area of 25 cm^2^.

**References**

[1] J. Kibsgaard, T. F. Jaramillo, *Angew. Chem. Int. Ed.* **2014**, *53*, 14433.

[2] X. Luo, H. Zhao, X. Tan, S. Lin, K. Yu, X. Mu, Z. Tao, P. Ji, S. Mu, *Nat. Commun.* **2024**, *15*, 8293.

[3] H. Y. Lin, Q. Q. Yang, M. Y. Lin, H. G. Xu, X. Tang, H. Q. Fu, H. Wu, M. Zhu, L. Zhou, H. Y. Yuan, S. Dai, P. F. Liu, H. G. Yang, *Adv. Mater.* **2024**, *36*, 2408045.

[4] T. Zhang, J. Jiang, W. Sun, S. Gong, X. Liu, Y. Tian, D. Wang, *Proc. Natl. Acad. Sci. U. S. A.* **2024**, *121*, e2317247121.

[5] S. Xu, S. Feng, Y. Yu, D. Xue, M. Liu, C. Wang, K. Zhao, B. Xu, J.-N. Zhang, *Nat. Commun.* **2024**, *15*, 1720.

[6] H.-J. Niu, N. Ran, W. Zhou, W. An, C. Huang, W. Chen, M. Zhou, W.-F. Lin, J. Liu, L. Guo, *J. Am. Chem. Soc.* **2025**, *147*, 2607.

[7] Y. Shi, L. Song, Y. Liu, T. Wang, C. Li, J. Lai, L. Wang, *Adv. Energy Mater.* **2024**, *14*, 2402046.

[8] S. Zhou, H. He, J. Li, Z. Ye, Z. Liu, J. Shi, Y. Hu, W. Cai, *Adv. Funct. Mater.* **2024**, *34*, 2313770.

[9] Y.-F. Cui, S.-D. Jiang, Q. Fu, R. Wang, P. Xu, Y. Sui, X.-J. Wang, Z.-L. Ning, J.-F. Sun, X. Sun, A. Nikiforov, B. Song, *Adv. Funct. Mater.* **2023**, *33*, 2306889.

[10] Q. Li, W. Luo, X. Cui, J. Shi, *Angew. Chem. Int. Ed.* **2025**, *64*, e202500303.

[11] W. Wu, Y. Wang, S. Song, Z. Ge, C. Zhang, J. Huang, G. Xu, N. Wang, Y. Lu, Z. Deng, H. Duan, M. Liu, C. Tang, *Angew. Chem. Int. Ed.* **2025**, *64*, e202504972.

[12] J. Sun, S. Zhou, Z. Zhao, S. Qin, X. Meng, C.-H. Tung, L.-Z. Wu, *Energy Environ. Sci.* **2025**, *18*, 1952.

[13] Y. Hu, T. Shen, Z. Wu, Z. Song, X. Sun, S. Hu, Y.-F. Song, *Adv. Funct. Mater.* **2025**, *35*, 2413533.

[14] Z. Cai, J. Liang, Z. Li, T. Yan, C. Yang, S. Sun, M. Yue, X. Liu, T. Xie, Y. Wang, T. Li, Y. Luo, D. Zheng, Q. Liu, J. Zhao, X. Sun, B. Tang, *Nat. Commun.* **2024**, *15*, 6624.

[15] Y. Chen, Q. Li, Y. Lin, J. Liu, J. Pan, J. Hu, X. Xu, *Nat. Commun.* **2024**, *15*, 7278.

[16] P. Ma, H. Cao, Q. Hao, R. Wang, W. Liu, M. Zuo, C. Jia, Z. Zhang, J. Bao, *Angew. Chem. Int. Ed.* **2024**, *63*, e202404418.

[17] Z. Li, Y. Yao, S. Sun, J. Liang, S. Hong, H. Zhang, C. Yang, X. Zhang, Z. Cai, J. Li, Y. Ren, Y. Luo, D. Zheng, X. He, Q. Liu, Y. Wang, F. Gong, X. Sun, B. Tang, *Angew. Chem. Int. Ed.* **2024**, *63*, e202316522.

[18] D. Li, R. Xiang, F. Yu, J. Zeng, Y. Zhang, W. Zhou, L. Liao, Y. Zhang, D. Tang, H. Zhou, *Adv. Mater.* **2024**, *36*, 2305685.

[19] S.-P. Zeng, H. Shi, T.-Y. Dai, Y. Liu, Z. Wen, G.-F. Han, T.-H. Wang, W. Zhang, X.-Y. Lang, W.-T. Zheng, Q. Jiang, *Nat. Commun.* **2023**, *14*, 1811.

[20] H. Zhang, H. Guo, Y. Li, Q. Zhang, L. Zheng, L. Gu, R. Song, *Adv. Funct. Mater.* **2023**, *33*, 2304403.

[21] Z. Liang, D. Shen, Y. Wei, F. Sun, Y. Xie, L. Wang, H. Fu, *Adv. Mater.* **2024**, *36*, 2408634.

[22] Z. Xiao, Y.-C. Huang, C.-L. Dong, C. Xie, Z. Liu, S. Du, W. Chen, D. Yan, L. Tao, Z. Shu, G. Zhang, H. Duan, Y. Wang, Y. Zou, R. Chen, S. Wang, *J. Am. Chem. Soc.* **2020**, *142*, 12087.

[23] A. K. Tomar, U. N. Pan, N. H. Kim, J. H. Lee, *ACS Energy Lett.* **2023**, *8*, 565.

[24] K. Dastafkan, S. Wang, C. Rong, Q. Meyer, Y. Li, Q. Zhang, C. Zhao, *Adv. Funct. Mater.* **2022**, *32*, 2107342.

[25] K. Wei, Z. Liu, G. Feng, Y. Wang, S. Zhang, X. Li, M. Zhang, H. Li, J. Zhou, S. Lei, F. Gao, *Adv. Energy Mater.* **2025**, *15*, 2405366.

[26] Q. Huang, C. Sun, D. Xie, J. Wang, B. Huang, D. Wen, D. Lin, C. Xu, W. Wu, T. Qiu, J. Wu, G.-J. Xia, Y. Wang, F. Xie, W. Guo, *Adv. Funct. Mater.* **2025**, 2506007.

[27] L. Zhang, N. Zhang, H. Shang, Z. Sun, Z. Wei, J. Wang, Y. Lei, X. Wang, D. Wang, Y. Zhao, Z. Sun, F. Zhang, X. Xiang, B. Zhang, W. Chen, *Nat. Commun.* **2024**, *15*, 9440.

[28] L. Guo, J. Chi, T. Cui, J. Zhu, Y. Xia, H. Guo, J. Lai, L. Wang, *Adv. Energy Mater.* **2024**, *14*, 2400975.

[29] H. Hu, Z. Xu, Z. Zhang, X. Yan, X. Wang, Y. Zhu, J. Wang, M. Yang, *Adv. Funct. Mater.* **2024**, *34*, 2403863.

[30] D. Cao, Z. Zhang, Y. Cui, R. Zhang, L. Zhang, J. Zeng, D. Cheng, *Angew. Chem. Int. Ed.* **2023**, *62*, e202214259.

[31] H. Lei, Q. Wan, S. Tan, Z. Wang, W. Mai, *Adv. Mater.* **2023**, *35*, 2208209.

[32] H.-J. Liu, S. Zhang, W.-Y. Yang, N. Yu, C.-Y. Liu, Y.-M. Chai, B. Dong, *Adv. Funct. Mater.* **2023**, *33*, 2303776.
